# Fertilization regime changes rhizosphere microbial community assembly and interaction in *Phoebe bournei* plantations

**DOI:** 10.1007/s00253-024-13106-5

**Published:** 2024-07-12

**Authors:** Haoyu Yan, Yang Wu, Gongxiu He, Shizhi Wen, Lili Yang, Li Ji

**Affiliations:** School of Forestry, Central South University of Forestry and Technology, 410004 Changsha, People’s Republic of China

**Keywords:** *Phoebe bournei*, Fertilization regimes, Microbial community, Community assembly, Co-occurrence network

## Abstract

**Abstract:**

Fertilizer input is one of the effective forest management practices, which improves soil nutrients and microbial community compositions and promotes forest productivity. However, few studies have explored the response of rhizosphere soil microbial communities to various fertilization regimes across seasonal dynamics. Here, we collected the rhizosphere soil samples from *Phoebe bournei* plantations to investigate the response of community assemblages and microbial interactions of the soil microbiome to the short-term application of four typical fertilizer practices (including chemical fertilizer (CF), organic fertilizer (OF), compound microbial fertilizer (CMF), and no fertilizer control (CK)). The amendments of organic fertilizer and compound microbial fertilizer altered the composition of rhizosphere soil bacterial and fungal communities, respectively. The fertilization regime significantly affected bacterial diversity rather than fungal diversity, and rhizosphere fungi responded more sensitively than bacteria to season. Fertilization-induced fungal networks were more complex than bacterial networks. Stochastic processes governed both rhizosphere soil bacterial and fungal communities, and drift and dispersal limitation dominated soil fungal and bacterial communities, respectively. Collectively, these findings demonstrate contrasting responses to community assemblages and interactions of rhizosphere bacteria and fungi to fertilizer practices. The application of organic fertilization strengthens microbial interactions and changes the succession of key taxa in the rhizosphere habitat.

**Key points:**

• *Fertilization altered the key taxa and microbial interaction*

• *Organic fertilizer facilitated the turnover of rhizosphere microbial communities*

• *Stochasticity governed soil fungal and bacterial community assembly*

**Supplementary Information:**

The online version contains supplementary material available at 10.1007/s00253-024-13106-5.

## Introduction

Fertilizer application is considered to be an essential forest management practice for improving soil fertility and forest productivity (Consalter et al. 2021). Fertilization practices based on scientific evidence can promote the growth and development of soil microbes, change soil properties, and thus facilitate the nutrient cycling capacity in the rhizosphere system (Sabir et al. 2021). Despite its high nutrient content and effectiveness, chemical fertilizer causes soil acidification and compaction (Mahmud et al. 2021). On the contrary, organic fertilizer can provide carbon-based nutrients and improve soil organic matter, but the shortcoming is the slow fertilization effect and the high oxygen consumption (Dincă et al. 2022). Biofertilizer is an environment-friendly, alternative fertilizer, which contains living microorganisms that can be fed to the rhizosphere to accelerate microbial processes and increase soil nutrient availability. It maintains the balance and health of the soil ecosystem (Mahanty et al. 2017). However, to date, much uncertainty still exists about the rhizosphere soil microbial community responsiveness to various fertilizer practices in forest ecosystems.

The rhizosphere plays a critical role as the engine of the soil ecosystem, which is the hotspot for interactions between plants and microbes, with strong spatial and temporal heterogeneity, and can accelerate the turnover of C and nutrients (Kuzyakov and Blagodatskaya 2015; Geisseler et al. 2017). The interactions of rhizosphere microbial members can improve soil physical properties, and soil fertility levels (Gong et al. 2019), regulate community assembly and community composition, as well as enhance stress resistance and improve disease prevention in forest trees (Dincă et al. 2022). The addition of organic fertilizer-enriched beneficial rhizosphere bacteria and bio-organic fertilizer manipulated the rhizosphere fungal community to inhibit the taxa of pathogenic fungi (Zhang et al. 2022). Numerous studies on acidic soils have shown that fungal community structure rather than fungal community diversity responded to fertilization (Ye and Lin 2020). The application of organic fertilizer increased bacterial diversity and altered the composition of the bacterial community (Gu et al. 2019; Li et al. 2015), and long-term fertilization further drove the change in bacterial community function (Li et al. 2015). Nevertheless, the mechanisms of fertilizer application on microbial interactions are still unclear, and the shift of key taxa under various fertilization regimes remains speculative. Network analysis can provide insights into identifying potential biotic interactions, niche overlap, and differentiation (Barberán et al. 2012). It was reported that organic fertilizer increased the complexity and connectivity of bacterial networks and reduced the interspecific competition of bacteria (Zhao et al. 2023). Moreover, regardless of abundance, network topological properties and the detection of potential keystone taxa supply detailed information about the community structure and function (Banerjee et al. 2018; Gao et al. 2022). Wang et al. (2022) found that the application of chemical fertilizers weakened the complexity of fungal networks and manure addition altered the keystone taxa of the fungal network. Therefore, unraveling the response of rhizosphere microorganisms to fertilization regime contributes to our knowledge of improving forest yield and mediating sustainable forestry.

Disentangling the mechanism of community assembly is critical for improving knowledge of the composition, structure, and function of rhizosphere soil microbiota (Wang et al. 2013; Huang et al. 2019; Jiao et al. 2020). Deterministic and stochastic processes are extensively considered important components in constructing community and maintaining species composition (Zhou and Ning 2017). Determinism is mainly appointed by environmental filtering and biological interactions (Graham and Knelman 2023), while stochasticity refers to ecological drift, probabilistic dispersal, and diversification that cause divergence during community succession (Dini-Andreote et al. 2015; Zhou and Ning 2017). The community is more susceptible to drift (stochastic process) or founder effects with lower biomass and smaller populations, and stochastic processes that contribute to the formation of species assemblages may be well-defined by the high level of biodiversity (Xun et al. 2019). Compared with bacteria, soil fungi typically exhibit larger sizes and slower growth rates, showing a preference for stable substrates. They are characterized by limited propagation and a higher resilience to dry conditions (Sun et al. 2017). In contrast, bacteria are significantly smaller, approximately one-tenth the size of fungi. They are known for their rapid growth rates, strong affinity for substrates, and their ability to diffuse easily, adapting swiftly to environmental changes (Rousk and Bååth 2007). In soil systems, bacteria and fungi support the size–plasticity hypothesis (Jiao et al. 2020; Isabwe et al. 2022). The strength of environmental selection generally decreases with the size of soil organisms, with smaller organisms being less filtered by the environment (Wu et al. 2018). Bacteria have a higher environmental tolerance than fungi, showing a stronger dispersal pattern (Isabwe et al. 2022). Communities driven by dispersal limitation tend to have higher microbial co-occurrence associations (Jiao et al. 2020). A global meta-analysis indicated that bacteria are greatly influenced by stochastic processes, while fungi are mostly structured by deterministic processes based on selection (Luan et al. 2020). Accumulating evidence has demonstrated that environmental variations may induce the shift of stochastic and deterministic processes under fertilization treatments (Stegen et al. 2012; Feng et al. 2018; Wan et al. 2021; Dong and Liu 2021). Sun et al. (2022) proposed that chemical fertilization can induce environmental filtration, while organic fertilization can push the stochastic process of the soil bacteria community. Jiao et al. (2022) revealed that soil fungal abundance mediates the balance of bacterial community assembly processes, and an increase in fungal abundance leads to a decrease in stochasticity. Soil bacterial communities under organic fertilizer treatment were less constrained by the environment and were governed by dispersal limitation (Wan et al. 2021). Additionally, a recent study has proven that seasonal dynamics of fungal community assembly are stronger than those of bacteria and protists (Yu et al. 2023). Nevertheless, far too little attention has been paid to the responses of ecological processes of rhizosphere soil microbiota to various fertilizer practices along with time. Unraveling the key processes involved in the community assembly of rhizosphere soil microbiota will contribute to a deeper understanding of the role of the interactions of plants and microorganisms in promoting soil fertilizer and forest productivity.

*Phoebe bournei*, as an evergreen broad-leaved and exclusive tree species in the subtropical region of China, is characterized by high ornamental and economic values (Luo et al. 2023a). Given the status of exhausted resources due to excessive logging by farmers and urgent demand from the market, *P. bournei* has been considered a national second-class rare and endangered tree species in China (He et al. 2022; Luo et al. 2023a). Sustainable production and high-quality cultivation of *P. bournei* plantations are urgently needed. Our previous findings confirmed that root exudates are more closely linked to soil microbial communities than soil physicochemical properties, as well as that functional information of soil bacteria and fungi exhibits higher resolution than a taxonomy (Luo et al. 2023a). In this study, we employed high-throughput sequencing technology to investigate the responses of ecological processes and interactions of rhizosphere soil microbiota to various fertilization practices combined with seasonal dynamics in the *P. bournei* young plantations. This study aimed to decipher the potential mechanisms of the divergent responses from the rhizosphere soil microbial communities towards various fertilization applications, which is highly significant for the rational optimization of rhizosphere process under fertilizer application. We tested the following hypotheses: (1) soil bacterial and fungal communities will respond differentially to different fertilization regimes and seasons. Specifically, organic fertilizers contribute more to the bacterial community, while compound microbial fertilizers interacte more dramatically with the fungal community. Fungi will be more sensitive to fluctuations in dry versus wet seasons than bacterial communities; (2) fertilization amendments will change the microbial interactions of rhizosphere soil bacteria and fungi; (3) stochasticity will dominate the ecological processes of rhizosphere soil bacteria and fungi, with fungi will dominate by drift processes and bacteria dominated by dispersal limitations, and bacterial and fungal communities exhibited ecological niche differentiation.

## Materials and methods

### Site description and experimental design

This study was conducted at the Jindong Forestry Farm located in Hunan Province, China (112°04′30″E, 26°18′30″N). With an average annual precipitation of 1745 mm, a temperature range of 18°C, and a frost-free duration of 260–344 days, this region experiences a humid subtropical monsoon climate. The two extremes are, respectively, 40 °C (July) and − 4.9 °C (January). *Phoebe bournei*, *Bretschneidera sinensis*, *Cunninghamia lanceolata*, *Cinnamonum camphora*, *Cephalotaxus oliveri*, *Ginkgo biloba*, *Ormosia henryi*, *Pinus massoniana*, *Pseudotsuga menziesii*, *Taxus chinensis*, and *Ilex chinensis* make up the typical vegetation in this region (Luo et al. 2023a). According to (United States Department Of Agriculture) USDA taxonomy (Soil Survey Staff 2022), the soil type is classified as Acrisol.

Five-year-old *P. bournei* seedlings with a planting density of 2.5 m × 2.5 m were planted in the spring of 2013 to establish a single plantation. In August 2018, three replicated plots (20 m × 20 m) of *P. bournei* young plantations were selected for each fertilization regime, including (1) no fertilizer (CK), (2) chemical fertilizer (CF) with 1000 g of N-P-K chemical fertilizer per tree (N:P:K = 15:15:15), (3) organic fertilizer (OF), which included 45% organic matter and 5% N, P, K, and (4) compound microbial fertilizer (CMF) with 20 g per tree amendment (more than 2 × 10^10^ of *Bacillus* per gram of fertilizer). Fertilizer was applied via the manual ring method (Luo et al. 2023a), and fertilizer from each plot was equally spread into the 0–10-cm soil layer after being precisely weighted.

### Soil sampling

The eight individual *P. bournei* trees were selected at random for sampling in each plot after 180 days of the fertilization amendment. Soil samples were collected in April, July, and October of 2019 and the following January. After the removal of stones and roots, rhizosphere soil was extracted from the 0–20-cm soil layer using a hand-held drill. Soil samples from each plot were pooled and homogenized. The collected samples were taken back to the laboratory and stored at − 80 °C for DNA extraction.

### DNA extraction and PCR amplification

Briefly, with the assistance of the MoBio PowerSoil DNA separation kit (MoBio Laboratories Inc., Carsbad, CA, USA), about 1 g (wet weight) of soil had its DNA extracted. The ITS1F (5′-CTTGGTCATTTAGAGGAAGTAA-3′) and ITS2R primers (5′-GCTGCGTTCTTCATCGATGC-3′) were used to amplify the ITS1 region, which is ubiquitous to all fungi (Luo et al. 2023a). Using the primers 338F (5′-ACTCCTACGGGAGGCAGCAG-3′) and 806R (5′-GGACTACHVGGGTWTCTAAT-3′), the 16S rRNA gene from the universal V3–V4 region of bacteria was amplified. The detailed PCR procedure is referenced in our study (Ji et al. 2022). For each sample, three PCR results were then combined and purified. The Illumina MiSeq platform (Illumina, San Diego, CA, USA) was used to sequence parallel-tagged samples. Using the UPARSE pipeline (version 7.0.1090 http://drive5.com/uparse/), high-quality sequences were condensed into operational taxonomic units (OTUs) with 97% similarity (Edgar 2013); the chimeric sequences were subsequently removed with USEARCH chimera filtering commands (http://drive5.com/usearch/manual/singletons.html). The National Center for Biotechnology Information’s (NCBI) Sequencing Read Archive (SRA) database received the raw sequencing data of the bacteria and fungi under the BioProject ID: PRJNA974557 and PRJNA974503, respectively.

### Statistical analysis

Before analyzing all the data, we first used the Kolmogorov–Smirnov test by SPSS software (IBM SPSS Statistics, Chicago, IL, USA) to confirm the normality of the data distribution for alpha diversity indices, the relative abundance of dominant taxa, and functional compositions; to investigate how the fertilizer regimes and seasons affected the soil microbial composition and diversity; and use a two-way analysis of variance (ANOVA). The 0.05 level of significance for a significant difference between treatments was determined using Tukey's honest significance test, which was carried out in SPSS software. All data are showed with mean ± standard errors. To predict the potential ecological functions of bacteria, we used the FAPROTAX database (http://www.loucalab.com, Louca lab. Department of Biology, University of Oregon, Eugene, OR, USA) (Louca et al. 2016). The FUNGuild tool (http://www.stbates.org/ guilds/app.php) was used to determine the identity of fungal communities (Nguyen et al. 2016), the rhizosphere soil fungal community was divided into dominant trophic modes (pathogenic, symbiotic, and saprotrophic) via FUNGuild analyses (Luo et al. 2023a). The community composition of the soil bacteria and fungi was examined using the “vegan” package (Oksanen et al. 2012) of the R software (version 4.2, The University of Auckland, Auckland, New Zealand). The variation in microbial communities was visualized via principal coordinate analysis (PCoA) ordination based on the Bray–Curtis distance matrix in R. The “adonis” function of the “vegan” package in R was used to execute a similarity analysis (ANOSIM) aimed at statistically significant variations (999 permutations) in community composition under four fertilizer regimes.

The co-occurrence networks were generated and analyzed via the Molecular Ecological Network Analysis pipeline (http://ieg2.ou.edu/MENA/main.cgi), which implements the random matrix theory (RMT) to investigate how fertilization treatments affect the interaction of soil bacterial and fungal communities (Deng et al. 2012). Molecular ecological networks were constructed via sequences of microbial taxa, which were devoted to analyzing the variations in network structures of soil bacterial and fungal communities and could effectively explain the interactions within the communities (Barberán et al. 2012; Ji et al. 2021). To compare the network topological properties among varied fertilizer applications, an identical cutoff of 0.88 and 0.78 was employed to construct the bacterial and fungal networks (Tu et al. 2020), respectively. Choosing the same network size and the average number of links to generate 100 corresponding random networks by the MENA pipeline. Soil fungal and bacterial networks were visualized using Gephi software 0.10.1. (https://gephi.org/) (He et al. 2022). *Zi*-*Pi* diagrams drawn by Origin software were used to identify the main nodes (Deng et al. 2012). Previous studies have noted that all nodes with *Zi* ≥ 2.5 or *Pi* ≥ 0.62 are designated as key species (Tu et al. 2020). Specifically, the node regions are divided into peripheral nodes (*Zi* < 2.5, *Pi* < 0.62), connected nodes (*Zi* < 2.5, *Pi* ≥ 0.62), module centroids (*Zi* ≥ 2.5. *Pi* < 0.62), and network centroids (*Zi* ≥ 2.5, *Pi* ≤ 0.62) in four categories (Ji et al. 2021).

Based on the taxonomic abundance of microbial communities and phylogenetic trees, the relative contribution of stochastic and deterministic processes in community assembly was quantified via the phylogenetic normalized stochasticity ratio (pNST) (Stegen et al. 2012, 2013). With 0.5 as the cutoff value, pNST > 0.5 was considered the dominant value of the stochastic processes in the group, while pNST < 0.5 or below was considered the dominant value of the deterministic processes in the group. Dispersal-based stochastic ecological processes of community assembly were further quantified by using the beta nearest taxon indices (*β*NTI) and the Raup–Crick (RC_bray_) null model based on the Bray–Curtis model (Stegen et al. 2013; Ji et al. 2022). For the dataset used in this study, the values of *β*NTI < − 2 denote deterministic process dominance and slow turnover (i.e., homogeneous selection), the values of *β*NTI > 2 denote deterministic process dominance and swift turnover (i.e., variable selection), and the values of − 2 < *β*NTI < 2 denote no deviation and deterministic process dominance, respectively (Stegen et al. 2012). The relative effects of stochastic processes are further separated according to the Bray–Curtis-based Raup–Crick metric (RC_bray_) (Stegen et al. 2013; Graham and Stegen 2017), dispersal limitation (RC_bray_ > 0.95), homogenizing dispersal (RC_bray_ < − 0.95), and undominated processes (− 0.95 < RC_bray_ < 0.95). All indices abovementioned were calculated by using the “iCAMP” package in R (https://github.com/DaliangNing/iCAMP1) with the code provided by Ning et al. (2020). Neutral theory combined with niche-based theory can also describe the assembly of microbial communities. The former is rooted in a stochastic process and the latter in recognizing a deterministic process. The neutral theory hypothesizes that the loss and gain of microbial communities are in stochastic balance (Sloan et al. 2006; Zhou and Ning 2017). The neutral community model calculation code (https://github.com/Weidong-Chen-Microbial-Ecology/) was obtained in R from Chen et al. (2019). Niche-based theory supports deterministic factors shaping microbial community assembly, including interspecies interactions, environmental conditions, and so on (Wiens et al. 2010). With the script provided by Zhang et al. (2018), the niche breadth was calculated using the function “niche_ breadth” of the “spaa” package in R; then, the generalized species and specialized species were divided by the “EcolUtils” package in R.

## Results

### Taxonomic and functional compositions of rhizosphere soil bacteria and fungi

**Fig. 1:**
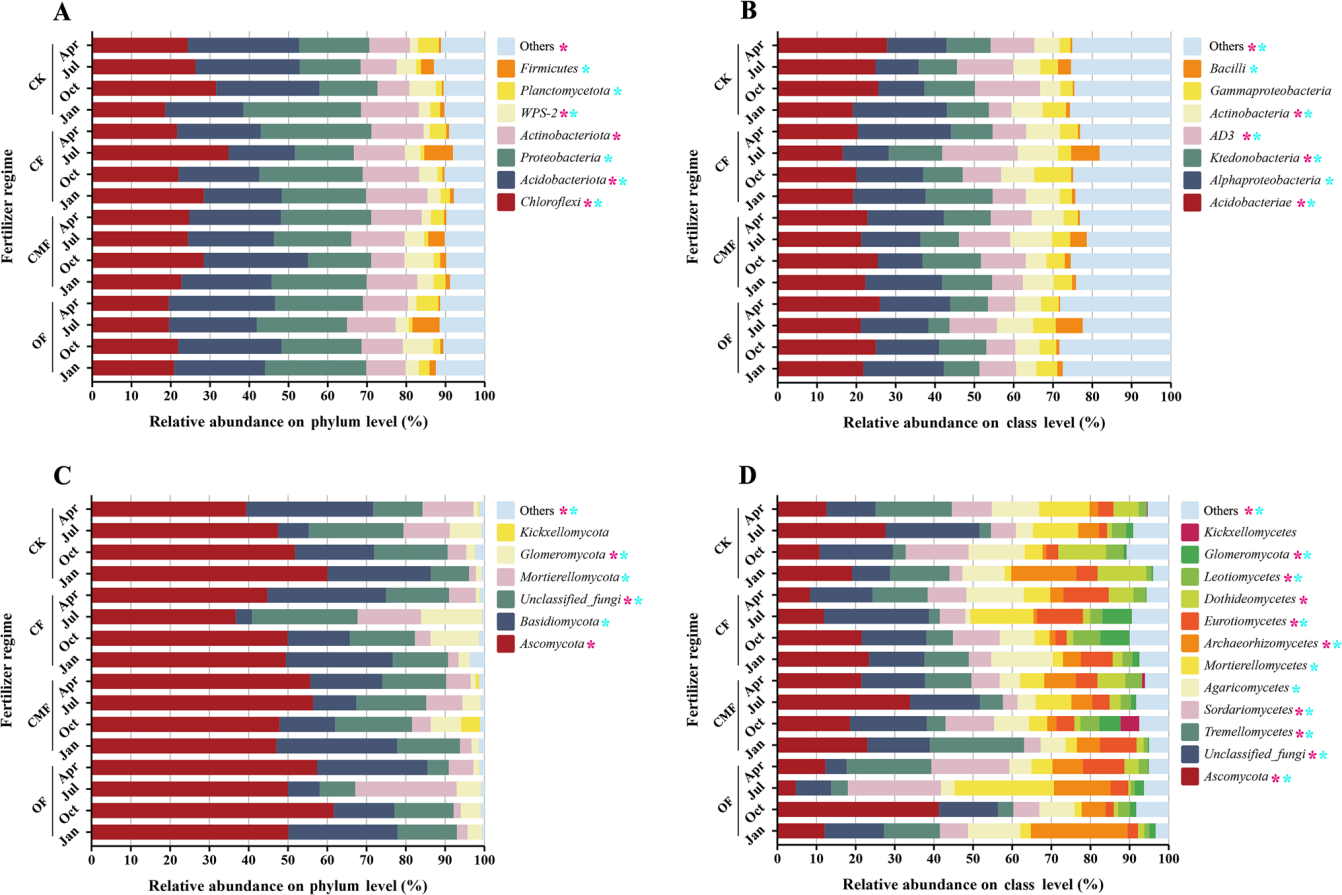
Relative abundances of dominant soil bacterial (**A**) and fungal (**C**) phyla under four fertilizer regimes across seasons. Relative abundances of dominant soil bacterial (**B**) and fungal (**D**) classes under four fertilizer regimes across seasons. CK, CF, OF, and CMF represent the control, chemical fertilizer, organic fertilizer, and compound microbial fertilizer, respectively. Low abundance phyla and classes with less than 0.5% of the total sequences across all samples are grouped into “Others.” The “*” with pink and blue indicate significant differences in the relative abundance of the microbial communities among fertilization regimes and seasons (*P* < 0.05), respectively

The high-quality sequences were categorized into 26 phyla and 54 classes within the soil bacterial community, 11 phyla, and 33 classes of soil fungal communities. The soil bacterial community was dominated by *Chloroflexi*, *Acidobacteriota*, *Proteobacteria*, and *Actinobacteriota* at the phylum taxonomic level, accounting for 81.3% of the total bacterial sequences (Fig. 1). In comparison to CK, the relative abundance of *Acidobacteriota* declined after fertilization, while the trend of relative abundance of *Proteobacteria* and *Actinobacteriota* rose (Fig. 1A). The CF regime significantly increased the relative abundance of *Actinobacteriota* (ranged from 5.7 to 78.6%) across seasons compared with CK, while the OF regime decreased the relative abundance of *Chloroflexi* (ranged from 25.8 to 30.5%) compared with CK. The relative abundance of *Chloroflexi* and *Proteobacteria* reached the highest and lowest levels in October and showed an opposite trend in January, respectively (Fig. 1A). The interaction between fertilization and season was strikingly significant for the relative abundance of *Chloroflexi* (*P* < 0.01). At the class level, the primary classes of the soil bacterial community were *Acidobacteriae* (22.5%), *Alphaproteobacteria* (16.9%), and others (24.5%), which comprised more than 60% of the total soil bacterial sequence (Fig. 1C). The significant impact of fertilization on the relative abundance of *Acidobacteriae* was greater than seasonal variation.

The dominant fungal community phyla were *Ascomycota* (50.4%) and *Basidiomycota* (19.8%). *Ascomycota* and *Glomeromycota* were significantly affected by the fertilization regime, and the relative abundance of *Ascomycota* under OF measure increased (ranged from 5.3 to 45.6%) compared with CK (Fig. 1B). The interaction between fertilizations and seasons had an appreciable impact on the fungal phyla except for *Agaricomycetes*, *Dothideomycetes*, *Leotiomycetes*, and *Kickxellomycetes*.

**Fig. 2:**
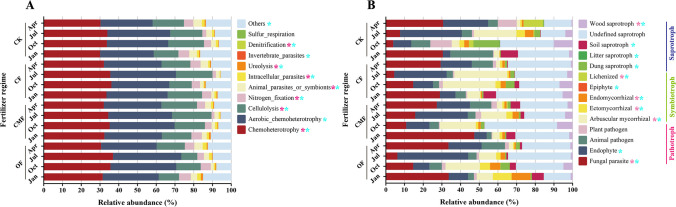
Relative abundances of dominant soil bacterial ecological function (**A**) and fungal guilds (**B**) under four fertilizer regimes across seasons. CK, CF, OF, and CMF represent the control, chemical fertilizer, organic fertilizer, and compound microbial fertilizer, respectively. The top 11 most abundant bacterial functions and the top 14 most abundant fungal functions were identified, respectively, and the rest of them are grouped into “Others.” The “*” with pink and blue indicate significant differences in the relative abundance of the microbial communities among fertilization regimes and seasons (*P* < 0.05), respectively

Based on the FRPROTAX database (http://www.loucalab.com, Louca lab. Department of Biology, University of Oregon, Eugene, OR, USA) (Louca et al. 2016), 4,365 soil bacterial OTUs were functionally annotated for functions related to the metabolism of substances such as C, N, and S elements. A total of 58 functions were detected in the rhizosphere soil bacterial sequences. The functional groups named chemoheterotrophy (33.4%), aerobic_chemoheterotrophy (32.1%), cellulolysis (15.7%), and nitrogen_fixation (4.3%) were the dominant ecological functions of bacteria and accounted for more than 80% of the total sequences (Fig. 2). Compared with CK, fertilization impacted the relative abundance of chemoheterotrophy, cellulolysis, nitrogen_fixation, animal_parasites_or_symbionts, intracellular_parasites, ureolysis, and denitrification substantially (*P* < 0.05) (Supplemental Table S1). Specifically, the relative abundance of chemoheterotrophy, nitrogen_fixation, and ureolysis under OF treatment increased by an average of 5.2%, 22.2%, and 46.5%, respectively. Compared with CK treatment, the relative abundance of cellulolysis under OF treatment decreased by ranging from 9.7 to 50.4% across seasons (Fig. 2A). Except for sulfur_respiration, the season had a strong effect on the ecological functions of the bacterial community.

It was found that the rhizosphere soil fungal community contained 14 functional guilds, which belong to pathotrophs, symbiotrophs, and saprotrophs. Pathotrophs accounted for the largest proportion (48.3%) of total sequences, followed by saprotrophs (34.9%) (Fig. 2). Compared with CK, CF treatment decreased the relative abundance of pathotrophs (19.3%) and increased the relative abundance of symbiotrophs (25.0%) and saprotrophs (19.7%). Fertilization significantly decreased the relative abundance of lichenized fungi, but significantly increased the relative abundance of arbuscular mycorrhizal fungi compared with CK. Overall, the interaction between fertilizations and seasons had remarkable results on all functional guilds (*P* < 0.05) (Supplemental Table S2), except for plant pathogen, endophyte, epiphyte, dung saprotroph, and wood saprotroph.

### Soil microbial α and β diversity under four fertilizer regimes

Fertilizer application dramatically affected the alpha diversity indices of rhizosphere soil fungal and bacterial communities (*P* < 0.01), except for the richness index of fungi (Table 1). In comparison with CK, the richness, Shannon, and Chao1 indices in the bacterial community increased significantly under the OF treatment. The richness and Chao1 indices of the fungal community were highest in the OF treatment, while the Shannon index was lowest and highest in the OF and CMF treatments, respectively. The highest values of richness and Chao1 indices under CK treatment were detected in April, whereas the lowest values were observed in October. Notably, the minimum values of fungal and bacterial community richness, Shannon index, and Chao1 index after fertilizer application were achieved earlier from October to July under the interaction of fertilizer application and season. Thus, with its seasonal variation, the soil microbial community showed a more divergent diversity in the wet season (April) than in the dry season (October).

**Table 1:** Richness, Shannon, and Chao 1 index of the bacterial and fungal community under four fertilizer treatments across seasons

Fertilizer regime	Season	Bacteria			Fungi		
		Richness	Shannon	Chao1 index	Richness	Shannon	Chao1 index
**CK**	**Apr**	1876 ± 47.57^Bb^	5.92 ± 0.04^Bb^	2590.73 ± 71.50^Bb^	838.33 ± 21.94^Aa^	4.32 ± 0.24^Aab^	989.89 ± 50.86^ABa^
	**Jul**	1875 ± 45.97^Bc^	5.92 ± 0.02^Bc^	2543.23 ± 59.64^Bc^	484.33 ± 18.50^Ac^	4.48 ± 0.03^Ab^	516.56 ± 31.36^ABc^
	**Oct**	1759 ± 59.56^Bd^	5.83 ± 0.06^Bb^	2267.02 ± 51.85^Bc^	402.33 ± 37.58^Ac^	4.83 ± 0.04^Aa^	426.16 ± 29.61^ABc^
	**Jan**	2210.33 ± 34.44^Ba^	6.23 ± 0.03^Ba^	3036.57 ± 138.77^Ba^	716.33 ± 95.71^Ab^	3.87 ± 0.27^Ab^	896.57 ± 129.04^ABb^
**CF**	**Apr**	1926.33 ± 56.70^Cb^	5.99 ± 0.15^Bb^	2668.33 ± 40.07^Cb^	837 ± 109.22^Aa^	4.21 ± 0.46^ABab^	983.57 ± 109.11^Ba^
	**Jul**	1522 ± 72.79^Cc^	5.45 ± 0.04^Bc^	2089.74 ± 156.60^Cc^	267 ± 35.55^Ac^	3.87 ± 0.27^ABb^	283.29 ± 42.77^Bc^
	**Oct**	1883 ± 40.85^Cd^	6.09 ± 0.14^Bb^	2458.24 ± 128.53^Cc^	516.67 ± 27.79^Ac^	4.69 ± 0.08^ABa^	539.62 ± 39.51^Bc^
	**Jan**	2008.33 ± 118.43^Ca^	6.12±0.10^Ba^	2752.5 ± 102.54^Ca^	722 ± 20.66^Ab^	4.38 ± 0.20^ABb^	812.13 ± 36.99^Bb^
**CMF**	**Apr**	1909 ± 69.86^BCb^	5.95 ± 0.07^Bb^	2665.44 ± 136.90^BCb^	939.33 ± 19.04^Aa^	4.67 ± 0.05^Aab^	1096.24 ± 13.80^ABa^
	**Jul**	1604 ± 59.35^BCc^	5.71 ± 0.10^Bc^	2181 ± 51.75B^Cc^	423 ± 32.36^Ac^	4.51 ± 0.11^Ab^	437.63 ± 33.65^ABc^
	**Oct**	1872.33 ± 108.45^BCd^	6.06 ± 0.12^Bb^	2463.66 ± 100.73^BCc^	404.67 ± 17.04^Ac^	4.41 ± 0.28^Aa^	457.13 ± 10.89^ABc^
	**Jan**	2073.67 ± 33.29^BCa^	6.18 ± 0.04^Ba^	2834.82 ± 54.26^BCa^	744 ± 75.02^Ab^	3.99 ± 0.22^Ab^	883.55 ± 111.90^ABb^
**OF**	**Apr**	2137 ± 71.71^Ab^	6.23 ± 0.05^Ab^	2905.39 ± 165.99^Ab^	791.33 ± 86.15^Aa^	4.01 ± 0.33^Bab^	1021.74 ± 115.57^Aa^
	**Jul**	1919.67 ± 58.35^Ac^	5.8 ± 0.15^Ac^	2661.95 ± 146.47^Ac^	473 ± 41.22^Ac^	3.91 ± 0.12^Bb^	516.17 ± 46.68^Ac^
	**Oct**	1942 ± 36.00^Ad^	6.18 ± 0.04^Ab^	2616 ± 109.10^Ac^	537 ± 50.09^Ac^	4.18 ± 0.43^Ba^	576.2 ± 67.33^Ac^
	**Jan**	2282 ± 61.26^Aa^	6.23 ± 0.09^Aa^	3143.77 ± 15.01^Aa^	725 ± 29.46^Ab^	4.18 ± 0.06^Bb^	864.66 ± 10.09^Ab^

CK, control; CF, chemical fertilizer; OF, organic fertilizer; and CMF, compound microbial fertilizer. Different uppercase letters and lowercase letters indicate statistically significant differences among the four fertilizer regimes and seasons according to the Tukey's test, respectively (*P* < 0.05)

**Fig. 3:**
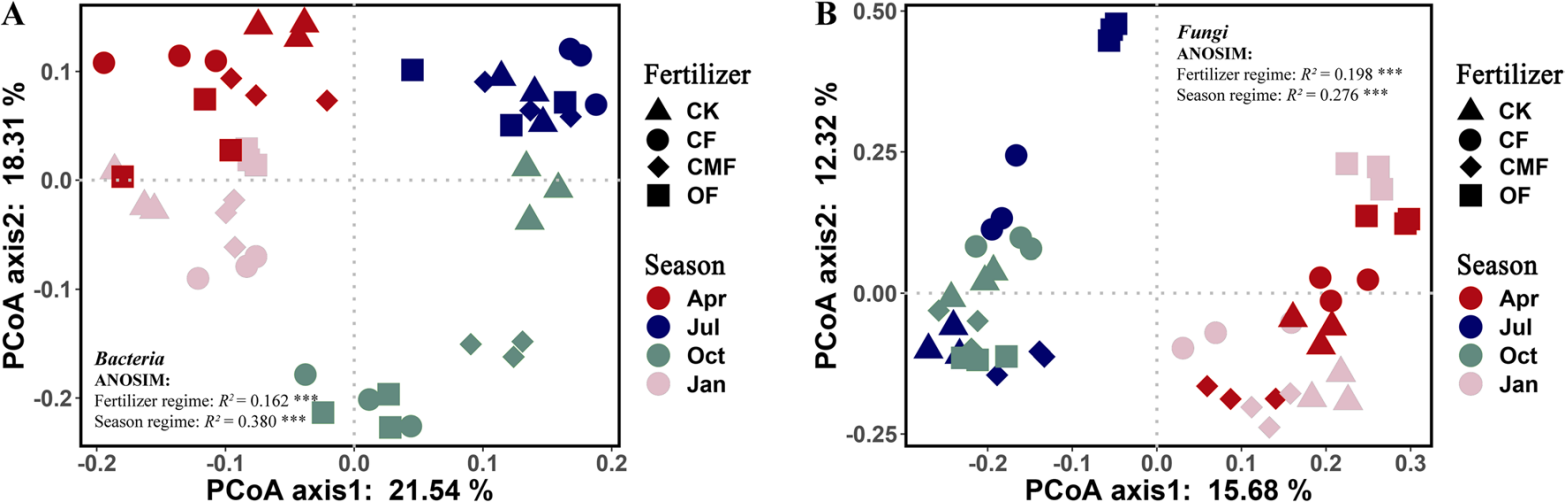
Principal coordinate analysis (PCoA) based on the Bray–Curtis dissimilarity matrices of bacterial (**A**) and fungal (**B**) communities. CK, control; CF, chemical fertilizer; CMF, compound microbial fertilizer; and OF, organic fertilizer. Apr, April; Jul, July; Oct, October; Jan, January. ***, *P* < 0.001. *R*^2^ represents the variation in bacterial and fungal community composition that can be explained by fertilizer regimes and seasons using ANOSIM

The differences in soil fungal and bacterial community compositions under various fertilization regimes and seasons were evaluated via PCoA. Both fertilizer regimes and seasons affected rhizosphere soil fungal and bacterial communities (Fig. 3). The rhizosphere soil bacterial and fungal communities could be explained by the values 39.9% (Fig. 3A) and 28.0% (Fig. 3B) from the first two axes, respectively. Moreover, the ANOSIM results showed that fertilization effects were significantly greater on fungal communities (*R*^2^ = 0.198) than on bacterial communities (*R*^2^ = 0.162), and bacterial communities (*R*^2^ = 0.380) experienced higher seasonal fluctuations than fungal communities (*R*^2^ = 0.276).

### Co-occurrence networks of rhizosphere soil bacteria and fungi

**Fig. 4:**
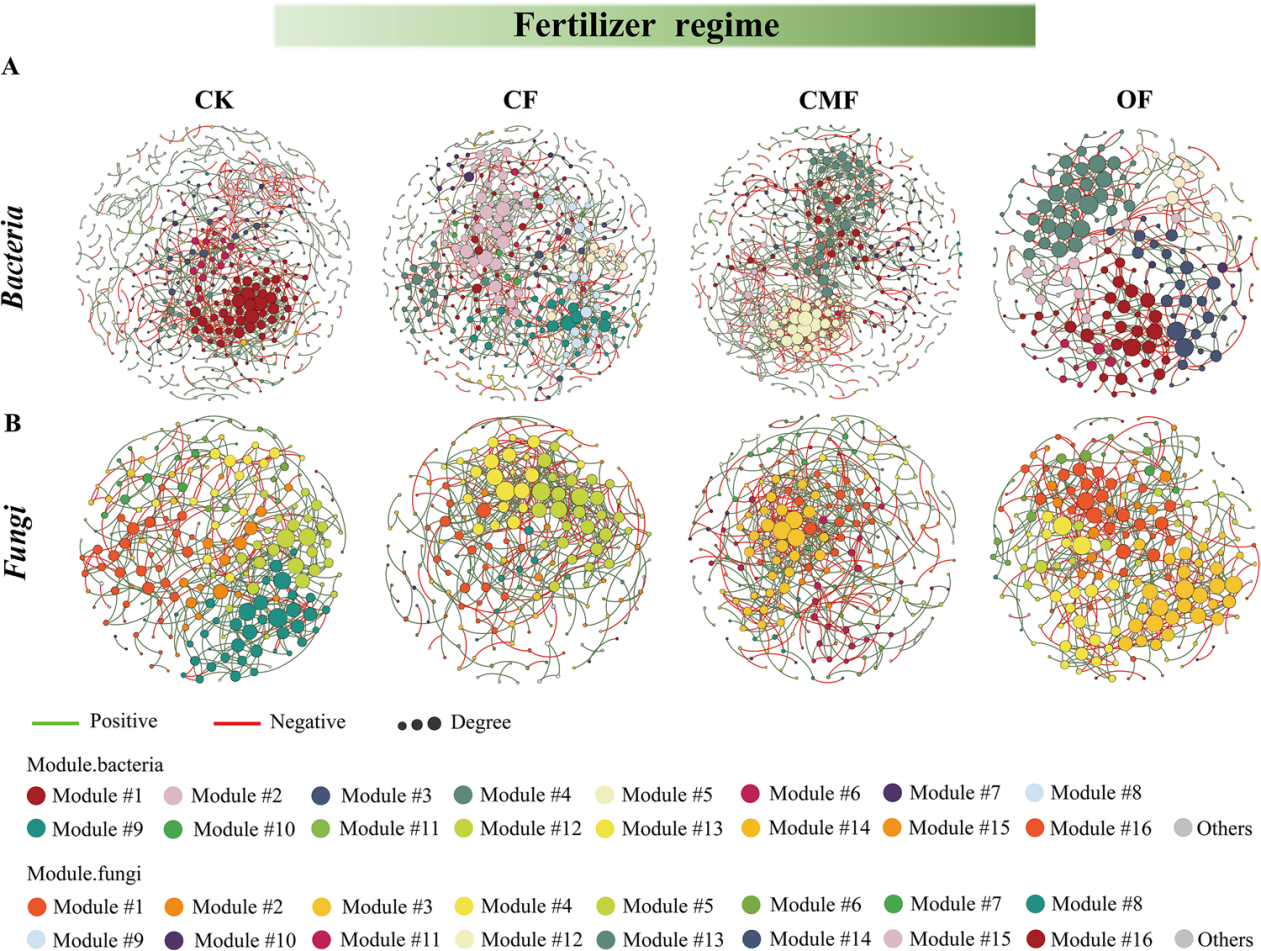
Modular networks of bacteria (**A**) and fungi (**B**) under four fertilizer regimes. Node colors represent different modules. The connections denote strong (Spearman’s *ρ* > 0.6) and significant (*P* < 0.01) correlations

Based on Spearman’s correlations, a total of 16 co-occurrence networks of soil fungal and bacterial communities under different fertilization management strategies (Fig. 4) across seasons (Supplemental Fig. S1) were constructed. For bacterial networks, the nodes, links, and average degree (avgK) of the network under the OF treatment were both decreased compared with the network of CK, and it formed a greater average geodesic distance, higher modularity, and the highest proportion of positive links (75.1%) (Table 2). The bacterial network under the CMF regime exhibited a tighter and more complex network structure compared with the network of CK, but the negative links increased. For fungal networks, positive interactions among fungal taxa after the amendment of fertilization regimes were obviously decreased compared with CK. More importantly, negative interactions under the OF regime floated the maximum, which resulted in a 15.59% increase in negative fungal correlations (Table 3). Meanwhile, the avgK and average clustering coefficient (avgCC) of the fungal network under CMF treatment decreased compared with CK, while the average path distance (GD) increased compared with CK. The rhizosphere fungal and bacterial networks had the lowest and highest proportion of positive interactions in October, respectively. Bacterial networks had lower avgCC, modularity, GD, and higher avgK than fungal networks in October. The changes in these network topological parameters implied that fertilizer regimes reinforced the competitive relationships among fungal community species. OF management had contributed much to the positive interactions of bacterial communities, and the CMF regime enabled the negative interactions of bacterial communities. The complexity of the bacterial network was higher than that of the fungal network.

**Table 2:** Topological properties of the co-occurrence network of bacteria

	Network features	CK	CF	CMF	OF	Apr	Jul	Oct	Jan
**Empirical network**	Number of nodes	484	477	478	470	456	417	399	418
	Number of links	800	795	928	719	569	529	974	479
	*R*^*2*^ of power-law	0.939	0.851	0.829	0.895	0.859	0.856	0.915	0.907
	Number of positive correlations	568 (71.0%)	583 (73.3%)	657 (70.8%)	540 (75.1%)	418 (73.5%)	396 (74.9%)	633 (65.0%)	313 (65.3%)
	Number of negative correlations	232 (29.0%)	212 (26.7%)	271 (29.2%)	179 (24.9%)	151 (26.5%)	133 (25.1%)	341 (35.0%)	166 (34.7%)
	Average degree (avgK)	3.306	3.333	3.883	3.060	2.496	2.537	4.882	2.292
	Average clustering coefficient (avgCC)	0.178	0.208	0.196	0.188	0.149	0.175	0.186	0.153
	Average path distance (GD)	8.573	7.026	6.565	8.394	8.149	8.128	4.738	6.507
	Harmonic geodesic distance (HD)	5.718	5.688	5.087	6.273	6.301	5.860	3.581	4.739
	Modularity	0.770	0.791	0.726	0.825	0.840	0.841	0.515	0.852
**Random network**	avgCC ± SD	0.013 ± 0.004	0.008 ± 0.003	0.016 ± 0.004	0.008 ± 0.003	0.005 ± 0.003	0.006 ± 0.003	0.053 ± 0.006	0.005 ± 0.003
	GD ± SD	4.485 ± 0.056	4.799 ± 0.055	4.142 ± 0.045	4.895 ± 0.065	5.859 ± 0.112	5.547 ± 0.111	3.494 ± 0.041	5.880 ± 0.159
	Modularity ± SD	0.576 ± 0.006	0.581 ± 0.007	0.509 ± 0.006	0.615 ± 0.006	0.715 ± 0.006	0.700 ± 0.008	0.402 ± 0.005	0.752 ± 0.007

**Table 3:** Topological properties of the co-occurrence network of fungi

	Network features	CK	CF	CMF	OF	Apr	Jul	Oct	Jan
**Empirical network**	Number of nodes	211	186	231	213	340	147	181	315
	Number of links	464	456	493	452	1009	260	376	821
	*R*^*2*^ of power-law	0.627	0.861	0.852	0.739	0.840	0.722	0.692	0.681
	Number of positive correlations	368 (79.3%)	336 (73.7%)	331 (67.1%)	288 (63.7%)	639 (63.3%)	179 (68.9%)	275 (73.2%)	544 (66.3%)
	Number of negative correlations	96 (20.7%)	120 (26.3%)	162 (32.9%)	164 (36.3%)	370 (36.7%)	81 (31.2%)	101 (26.9%)	277 (33.7%)
	Average degree (avgK)	4.398	4.903	4.268	4.244	5.935	3.537	4.155	5.213
	Average clustering coefficient (avgCC)	0.233	0.202	0.194	0.245	0.237	0.216	0.223	0.226
	Average path distance (GD)	5.011	4.819	5.405	4.844	4.692	5.456	4.888	4.802
	Harmonic geodesic distance (HD)	4.078	3.630	4.293	4.015	3.954	4.197	3.931	4.035
	Modularity	0.664	0.539	0.613	0.656	0.554	0.680	0.641	0.639
**Random network**	avgCC ± SD	0.023 ± 0.006	0.050 ± 0.010	0.033 ± 0.007	0.024 ± 0.008	0.036 ± 0.005	0.025 ± 0.010	0.027 ± 0.008	0.022 ± 0.005
	GD ± SD	3.712 ± 0.043	3.366 ± 0.059	3.717 ± 0.062	3.756 ± 0.042	3.390 ± 0.030	3.938 ± 0.074	3.708 ± 0.049	3.616 ± 0.029
	Modularity ± SD	0.459 ± 0.009	0.403 ± 0.007	0.464 ± 0.008	0.468 ± 0.009	0.374 ± 0.005	0.518 ± 0.012	0.471 ± 0.008	0.414 ± 0.006

The rhizosphere soil bacterial (Supplemental Fig. S2A) and fungal (Supplemental Fig. S2B) networks under different fertilization regimes consisted of 35 and 30 keystone species, respectively. Fertilization changed the composition and sum of key species in bacterial and fungal networks. The keystone species of rhizosphere soil bacterial networks included 10 in *Acidobacteriota*, 7 in *Proteobacteria*, 5 in *Actinobacteriota*, and 4 in *Chloroflexi,* which occupied more than 70.2% of the total keystone species (Supplemental Table S3). Compared with CK, the OF regime enriched *Acidobacteriota*, *Chloroflexi*, and *Planctomycetota* key species and depleted *Actinobacteriota and Proteobacteria* key species in the bacterial network. *Bradyrhizobium*_OTU1317 from the bacterial network under CMF treatment was identified to have ecological functions such as aerobic_chemoheterotrophy, chemoheterotrophy, and nitrogen_fixation. There were 31 keystone species in the bacterial network across seasons (Supplemental Fig. S2C), and the most and least keystone species were detected in October (51.6%) and January (6.5%) (Supplemental Table S3), respectively. The keystone species from rhizosphere soil fungal networks (Supplemental Fig. S2B), contained 13 in *Ascomycota*, 7 in *Glomeromycota*, 6 in *Unclassified_fungi*, 1 in *Mortierellomycota*, 1 in *Chytridiomycota*, and 1 in *Basidiomycota* (Supplemental Table S4). The key taxa *Mortierellomycota* of fungal networks were detected in CMF treatment relative to CK. Under the CMF regime, the key species of the fungal network were assigned to wood saprotroph, endophyte, litter saprotroph, arbuscular mycorrhizal, soil saprotroph, and undefined saprotroph. *Scytalidium*_OTU1317 probably belonged to wood saprotroph. The most and least keystone species of fungal networks were found in April (20.0%) and July (55.0%), respectively.

### Community assembly processes of rhizosphere soil microbiota

**Fig. 5:**
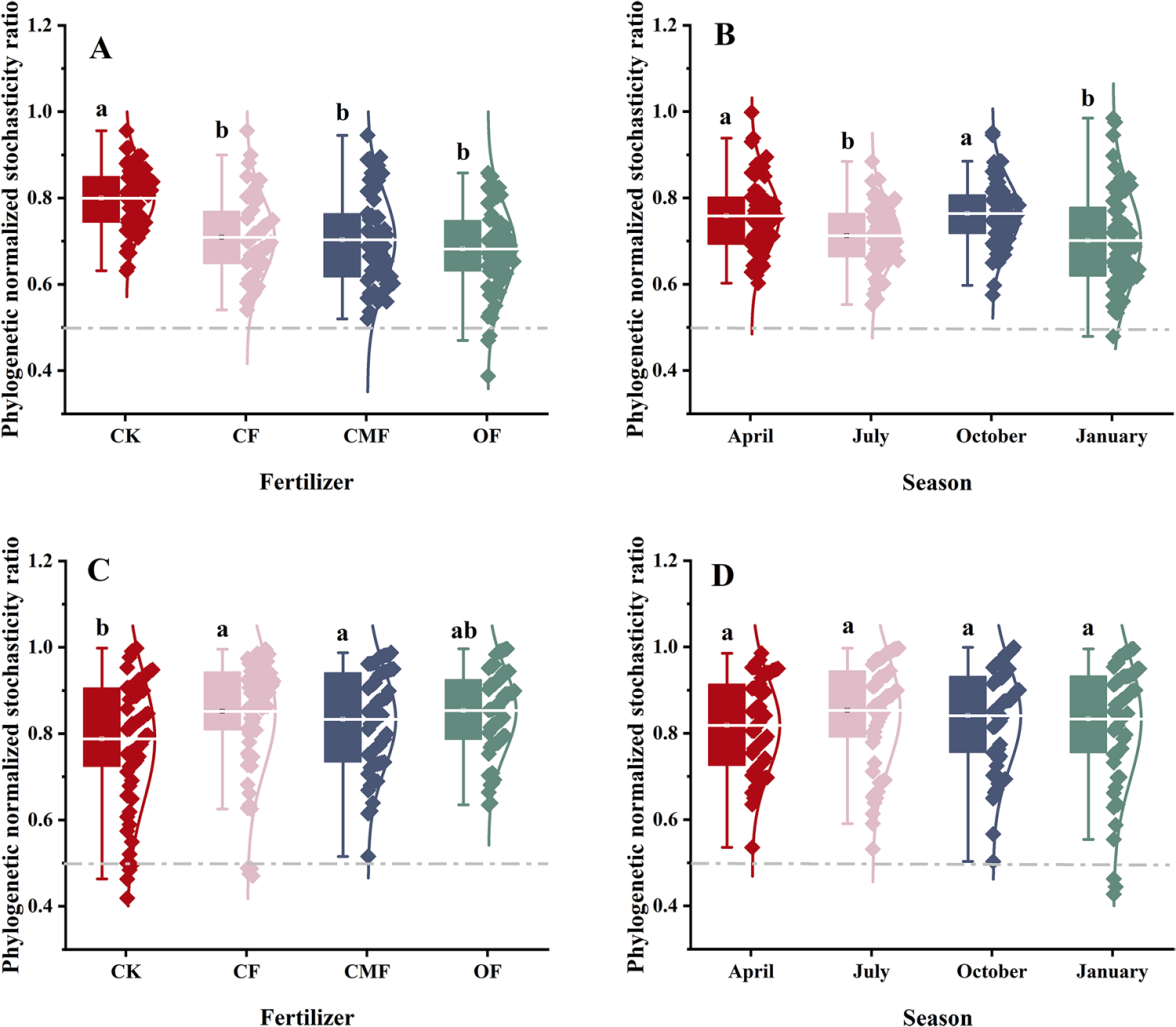
The ecological stochasticity in bacterial (**A**, **B**) and fungal (**C**, **D**) community assembly is estimated by the phylogenetic normalized stochasticity ratio (pNST). The value of 0.5 is the boundary point between more deterministic (< 0.5) and more stochastic (> 0.5) assembly. Different lowercase letters indicate the significant difference in the same regime (Tukey's test, *P* < 0.05)

**Fig. 6:**
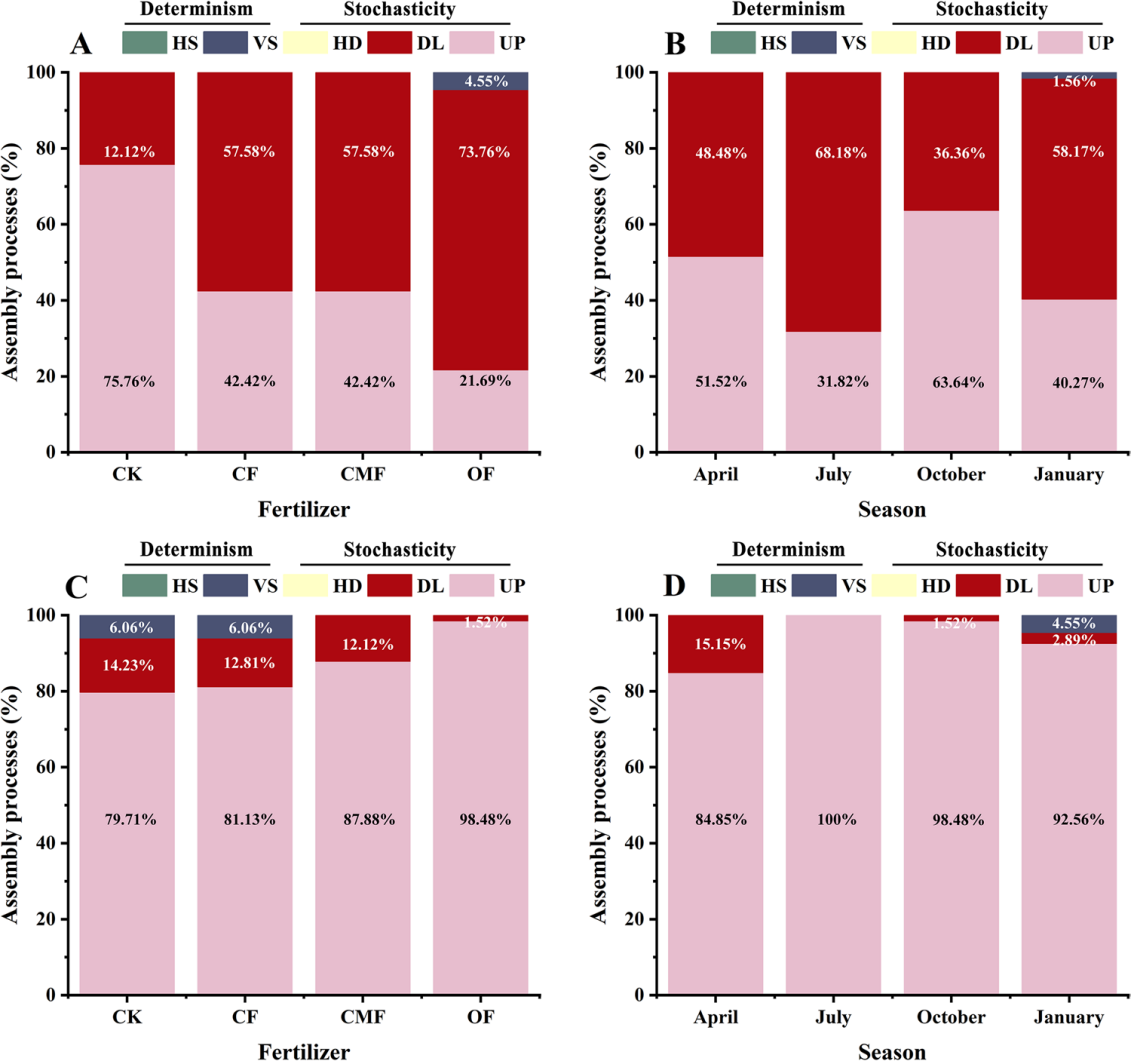
The relative contributions (%) of the bacterial (**A**, **B**) and fungal (**C**, **D**) community assembly processes based on pNST. HS, homogeneous selection; VS, variable selection; HD, homogenizing dispersal; UP, drift process; DL, dispersal limitation

The pNST values of rhizosphere soil fungal and bacterial communities under four fertilization regimes were mainly more than 0.5, which indicated that both soil fungal and bacterial communities were governed by stochastic processes (Fig. 5). Compared with CK, the amendment of fertilization increased and decreased the relative value of stochasticity in rhizosphere soil fungal and bacterial communities (Fig. 5A, B). Additionally, stochastic processes occupied the community assembly in soil bacteria and fungi across seasons. More importantly, in January, the determinism exhibited a few contributions to soil fungal and bacterial community assemblages (Fig. 5C, D). Specifically, the assembly of rhizosphere soil bacterial and fungal communities was predominately dominated by dispersal limitation and drift processes, respectively. For the rhizosphere soil bacterial community, the application of fertilization significantly increased the proportion of dispersal limitation (Fig. 6A, B). Compared with CK, under CF, CMF, and OF regimes, the proportions of dispersal limitation increased by 33.3%, 33.3%, and 49.5%, respectively (Fig. 6A). In contrast to the trend of bacterial community, the drift process of soil fungal community under the OF regime strikingly increased by 18.8% compared with CK (Fig. 6B).

Based on the neutral community model, under fertilization management strategies, bacterial communities were more dominated by neutral processes than fungal communities (Supplemental Fig. S3). The highest *R*-squared value and migration rate were detected under CK treatment compared with the other three fertilization regimes. Notably, fertilizer application improved the migration rate of the rhizosphere soil fungal community and alleviated the species dispersal limitation of soil fungi (Supplemental Fig. S3). More importantly, the migration rates of rhizosphere soil bacteria and fungi exhibit a consistent pattern along the seasonal dynamic; that is, higher values were found in April and January (Supplemental Fig. S3).

**Fig. 7:**
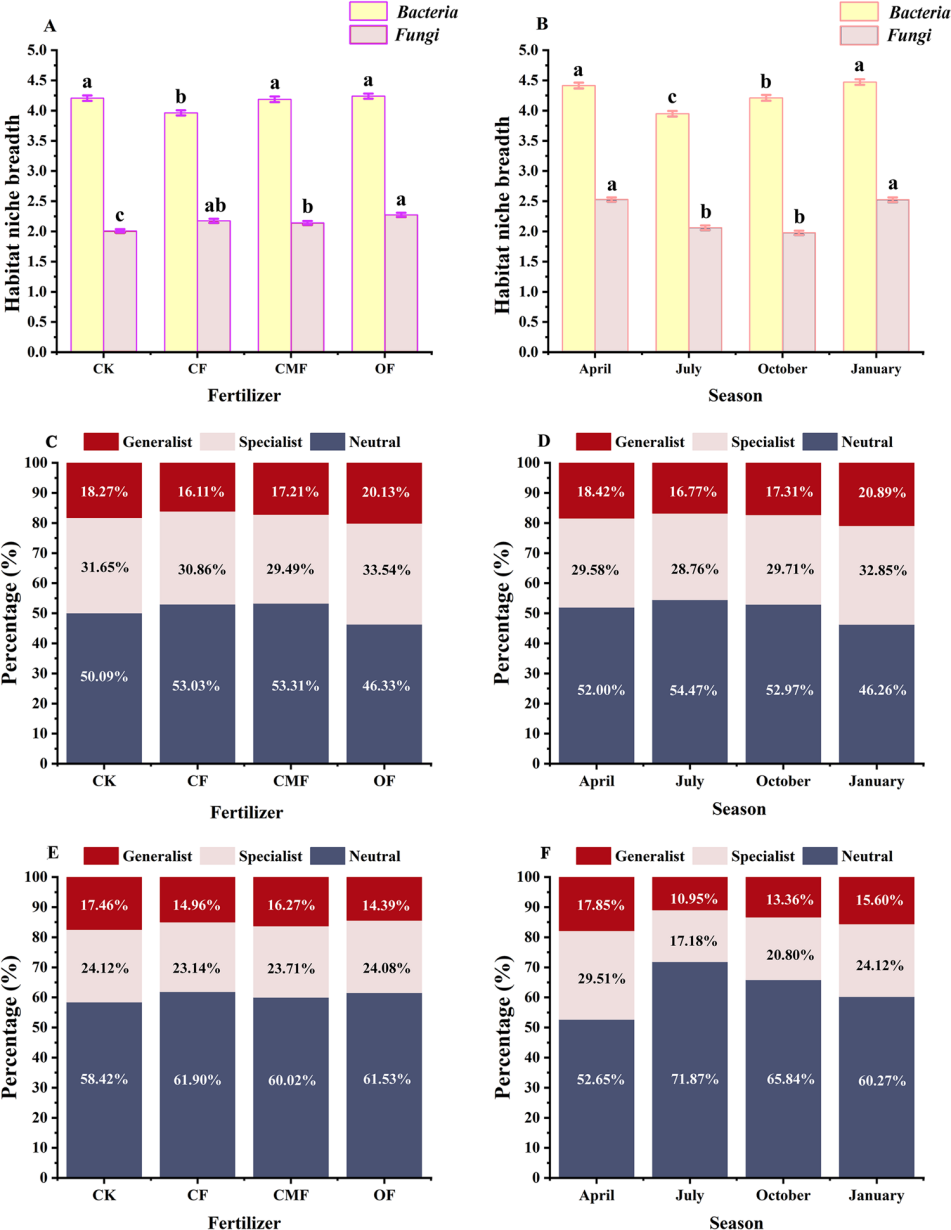
Niche breadth of bacteria and fungi under fertilizer regimes (**A**) across seasons (**B**). Different lowercase letters indicate the significant difference in the same regime (Tukey's test, *P* < 0.05). The rhizosphere bacterial (**C**, **D**) and fungal (**E**, **F**) communities were classified as generalized species, specialized species, and neutral species. OTUs with actual niche breadth indices exceeding the upper 95% confidence interval of the zero distribution were defined as generalist species, those below the lower 95% confidence interval of the zero distribution were defined as specialist species, and those within the 95% confidence interval of the zero distribution were defined as neutral taxa

The values of niche breadth of soil fungal communities under different fertilization regimes were lower than those of bacterial communities, indicating that soil bacteria had a preference for utilizing resources than fungi (Fig. 7A, B). The value of niche breadth of bacterial communities under CF decreased compared with CK (*P* < 0.05). There was no notable difference in soil bacterial niche breadth between the OF and CK treatments, while the OF practice increased the soil fungal niche breadth compared with CK (Fig. 7A). Compared with fertilization, both soil fungal and bacterial niche breadth exhibited obvious seasonal fluctuations, which showed a lower value in July and October (Fig. 7B). Both soil fungal and bacterial communities accounted for more specialist species than their respective generalist species under fertilization practices and seasons. For the rhizosphere soil bacterial community, compared with CK, the proportion of generalists and specialists increased under the OF treatment (Fig. 7C). The proportion of specialists in the rhizosphere fungal community showed no obvious differences compared with CK, while the proportion of generalists exhibited a remarkable upward trend (Fig. 7D). There was an obvious increase in generalists (32.9%) and specialists (20.9%) in the soil bacterial community in January (Fig. 7E). Nevertheless, the rhizosphere soil fungal community exhibited a similar trend in April (Fig. 7F).

## Discussion

### Fertilization changed bacterial α-diversity and season exhibited more significant effects on rhizosphere microbial β-diversity

As hypothesized, in the present study, organic fertilizers contributed more to the bacterial community, while compound microbial fertilizers interacted more dramatically with the fungal community. This result is consistent with our previous report (Luo et al. 2023a). Moreover, in agreement with other studies (Shi et al. 2020; Li et al. 2021; Ma et al. 2021), the OF treatment increased the relative abundance of *Proteobacteria*, *Actinobacteriota*, and *Planctomycetota*, and decreased the relative abundance of *Chloroflexi* and *Acidobacteriota* in contrast to CK. We may explain these findings by the divergent preferences of ecological strategies of rhizosphere soil bacterial trophic taxa. In agreement with previous studies, carbon source and nutrients provided by OF promoted copiotroph-dominated bacterial members (including *Proteobacteria* and *Bacteroidetes*) (Bei et al. 2018) and suppressed oligotrophs (mainly composed of *Acidobacteria* and *Chloroflexi*) (Ma et al. 2021; Luo et al. 2023a). Generally, oligotrophs were less competitive than copiotrophs because of their high nutrient content (Klarenberg et al. 2022). Liu et al. (2020) revealed that *Proteobacteria* and *Actinobacteriota* were strongly favored by nutrient inputs in a kiwifruit orchard plantation. The metabolism of *Proteobacteria* was accelerated in a nutrient-rich environment, which had been documented in previous research (Nielsen et al. 2014). *Actinobacteriota* and *Planctomycetes* were involved in decomposing organic matter (Ahn et al. 2012) and a variety of ecological processes in rhizosphere habitats (Shi et al. 2020). The relative abundance of *Acidobacteriota* and *Chloroflexi* was reduced because oligotrophs were characterized by lower growth rates and weaker substrate decomposition (Trivedi et al. 2013). The OF regime enhanced the relative abundance of aerobic_chemoheterotrophy, nitrogen_fixation, and ureolysis, and reduced the relative abundance of cellulolysis. There is growing evidence that OF can alter the function of soil bacterial and fungal communities by promoting saprophytic fungal synergies to break down more stable organic compounds such as lignin and humus (Ma et al. 2023). The CF regime was more effective than the OF regime in inhibiting the growth of pathotrophs and promoting the growth of saprotrophs; however, this result is not consistent with some studies. Ren et al. (2023) suggested that long-term organic fertilization regimes significantly reduced the relative abundance of fungal pathogens. It is widely demonstrated that saprotrophs also contribute to the suppression of fungal pathogens (Van Der Heijden et al. 2008). The results may be explained by the fact that CF is more effective in a short period due to the efficient fertilization effect compared with OF, whereas OF as a slow-release fertilizer would be more beneficial in suppressing fungal pathogens under long-term fertilization conditions.

Furthermore, our results showed that soil fungal and bacterial communities responded differently to season, which was in line with other previous similar studies (Zhao et al. 2018; Ma et al. 2021). Our observation revealed that *Actinobacteria* were more abundant in the dry season, while *Acidobacteria* were more abundant in the wet season. *Actinobacteria* members are known for their ability to withstand drought conditions (Pajares et al. 2018). Additionally, *Actinomycetes* increased in number during the dry season due to the decrease in rhizosphere secretions (Ma et al. 2021). The enrichment of *Acidobacteria* agreed with the described oligotrophic life-strategy of this phylum according to nutrition and growth-based classification (Fierer et al. 2007). For fungi, the relative abundance of *Ascomycota* decreased during the wet season but increased that of *Basidiomycota*. This finding is in agreement with a study conducted in a subtropical forest (Zhao et al. 2018) where the authors have suggested that the pattern could be attributed either to the taxa preference for moisture. The seasonal dynamics of fungal and bacterial diversity showed consistent patterns, that is, greater diversity in the dry season than in the wet season. This observation is inconsistent with earlier studies, which concluded that lower soil temperature leads to a decrease in microbial diversity (Dini-Andreote et al. 2014). In our study, the diversity indices of fungal and bacterial communities achieved peaks in April and January, respectively. The reason may be that the accumulation and decomposition of organic matter stimulate bacterial diversity due to the contribution of accumulated litter before January (Berg et al. 1998), and another explanation is that plants have a lower nutrient demand in January during the non-growing season, thereby enhancing bacterial diversity (Ma et al. 2021). Most of the changes in soil fungal communities occur on a longer time scale than bacteria, which is related to fungal life strategies (Sun et al. 2017).

The present findings revealed that fertilization significantly changed bacterial alpha diversity and the OF regime was effective in increasing the alpha diversity of bacterial communities, which coincided with the studies of Liu et al. (2020) and Li et al. (2022). However, the alpha diversity of fungal communities was not dramatically altered by fertilizer application. Ye and Lin (2020) highlighted that fungal diversity is more stable in acidic soils than in community structures after fertilization. Ding et al. (2017) found that organic fertilizer slightly increased the beta diversity of the fungal community during 35 years of experiments. Taken together, these findings confirmed that fertilization changed bacterial diversity but season had more profound effects on rhizosphere microbial diversity.

### Fertilization induced more negative interactions in fungi, but not in bacteria

Co-occurrence networks are increasingly being used to assess the potential mechanisms of microbial interspecific relationships (Faust and Raes 2012). As expected in our second hypothesis, fertilizer application altered the interactions of rhizosphere soil bacteria and fungi. Critically, organic fertilizer stimulated positive feedback from the bacterial network and negative feedback from the fungal network. The OF regime contributed to bacterial growth to a certain extent (Luo et al. 2023b), which, in turn, prompted bacterial networks to facilitate their mutual benefit. However, the interspecific competition for fungal networks was reinforced by limited resources (Ma et al. 2023). Our results proved that CMF treatment intensified intraspecific competition among bacterial and fungal taxa. *Bacillus* of CMF acted directly on the rhizome to stimulate bacterial and fungal communities to access resources, which could adjust the perception of bacterial and fungal competitors (Andrić et al. 2020). In addition, fungal networks were susceptible to precipitation and temperature changes caused by seasonal changes (Luo et al. 2023b). The result demonstrated that bacterial networks were the most stable in October and interspecific competition was the most intense. Fungal networks became less complex but highly modular in October and tended to cooperate between species. The reason for considering this result is that during the dry season when precipitation is low, fungal communities are more sensitive to precipitation change than bacterial communities (He et al. 2017), which tends to enhance the intensity of cooperative behavior among members and form higher modularity to resist such environmental disturbances (Liu et al. 2022). Overall, fertilization induced fungal networks that interacted negatively, while promoting bacterial networks that interacted positively. In addition, OF management promoted the intensity of cooperative behavior among bacterial network members, while strongly stimulating competition among fungal network members.

Keystone species are taxa with high connectivity in the co-occurrence network, which can drive the composition and function of microbial communities (Banerjee et al. 2018). Compared with CK, the OF treatment induced the emergence of *Pajaroellobacter* and *Gemmataceae* bacterial keystone species and CMF practice resulted in *Mortierellomycota* key species derived from fungal networks. *Pajaroellobacter* is capable of degrading lignin and cellulose using recalcitrant carbon (Fierer et al. 2007). *Gemmataceae* has been shown to promote root growth and be beneficial to bacterial diseases (Xiao et al. 2022). *Mortierellomycota* is a fast decomposer of rhizosphere fungi that secretes oxalic acid to dissolve phosphorus in the soil and increase soil nutrients (Wang et al. 2020). Moreover, several keystone species in the microbial community were identified to share OTU names. Similar studies have also reported that key species in microbial networks used the same OTU name to undertake the transformation of different roles (Lu et al. 2013). The key species with topological properties existed in overlapping niches in the microbial community (Faust and Raes 2012). We observed that *Bradyrhizobium*_OTU1317 and *Scytalidium*_OTU1317 play the critical role in microbial networks under the CMF regime. *Bradyrhizobium* is an important diazotrophic member with the capacity to fix substantial amounts of nitrogen, even in nitrogen-limited habitats (Tao et al. 2021). Most species of *Scytalidium* grow saprotrophically on wood (Quandt and Haelewaters 2021). Notably, we also observed low relative abundances of some keystone species such as OTU649, OTU3773, OTU87, and OTU1343. This indicates that keystone species do not necessarily need to be in high abundance, and a low abundance of keystone species could still provide greater connectivity for the community, which is conducive to enhancing microbial interactions (Herren and McMahon 2018). Recent studies have also revealed that low abundance or rare taxa play an important role in community functional diversity and ecosystem stability (Wang et al.2022b). Overall, fertilization altered the key taxa of microbial communities. Regardless of abundance, keystone species drive the community composition and function (Banerjee et al. 2018).

### The drift process dominated fungal community assembly, while dispersal limitation acting with drift governed bacterial community assembly

Our results highlighted that stochasticity drove the scale of much of the community assembly processes in bacteria and fungi, which supported our third hypothesis. The increase in soil fertility was reported to reinforce stochastic effects in microbial communities to weaken the effects of niche allocation (Yu et al. 2019). Dispersal limitation dominated the rhizosphere soil bacteria in community assembly after fertilization in this work. Previous studies revealed that fertilization increased the diversity of the bacterial community (Sabir et al. 2021) and thus enhanced the effect of dispersal limitation acting with drift (Chase 2010). In contrast, the fungal communities were controlled by drift after fertilization. Our finding is consistent with the study of Isabwe et al. (2022), highlighting the fact that soil bacteria occupy a wider niche than fungi, and bacteria are more easily dispersed than fungi. On the other hand, fungal communities and protists showed similar underlying mechanisms in the assembly process (Isabwe et al. 2022). Both dispersal and determinism shaped the fungal and bacterial communities in January, and fungal communities were more affected by variable selection than bacterial communities. Feng et al. (2018) confirmed that deterministic processes occurred under poor soil nutrient status, and yet stochastic assembly processes increased under superior nutritional conditions. The reason for considering this result is that in January, when plants grow in the non-growing season, root exudate and soil organic matter are depleted, and nutrient cycling with a lower level and environmental conditions increase the proportion of deterministic factors in the assembly process (Ma et al. 2021). Fungal communities are more subject to environmental filtering because bacteria are smaller than fungi, and smaller organisms have a higher metabolic capacity and greater environmental tolerance (Wu et al. 2018).

The null model elucidated the divergence in fungal and bacterial communities’ response to their respective community assembly processes after the application of fertilizer. The combination of neutral model analysis and niche breadth results further confirmed that the application of OF mobilized synergistic relationships between bacteria and fungi, with a propitious trend for community turnover. Bacterial communities had a greater niche breadth and more generalists under the OF treatment than fungal communities, indicating that bacteria had potentially higher phenotypic plasticity than fungi (Zhang et al. 2021). Organic fertilizer can easily cause environmental heterogeneity (Zhou and Ning 2017) and induce community assembly to be affected by determinism (Liu et al. 2023). Meanwhile, the fungal community transformed to be dominated by drift under the OF management, and OF formed a broader niche breadth and a higher species migration rate. Therefore, there was an obvious difference in altering the community assembly processes after the application of OF, and the OF regime was more conducive to community turnover between fungal and bacterial communities.

In addition, the results of fungal and bacterial community response to seasons indicate that dry conditions were more favorable for bacterial communities to capture resources, which weakened the competition of fungal communities. Correspondingly, wet conditions prompted fungal communities to be more sensitive to ecological factors and tended to give fungal communities sufficient resources. This lopsided response may be explained in part by differences in the adaptations of fungi and bacteria (Harris 2015). Soil bacterial community structure has stronger adaptability to moist conditions (Fierer et al. 2003). Our results are also supported by the research of He et al. (2017) on subtropical forests in China. In turn, the complementary niche breadth of fungal and bacterial communities in seasons makes them dependent on each other and weakens competition, which is more profitable to the stability of microbial communities. Chen et al. (2019) revealed that stochastic processes dominate the construction of subtropical planktonic communities in the wet and dry seasons and that the relative contribution of stochastic processes to microbial communities is greater in the wet season. Consequently, the present findings demonstrate that, in our study, fungal community assembly was governed by drift, while bacterial community assembly was controlled by dispersal limitation acting in conjunction with drift.

In summary, this study provides insights into the divergent response of soil bacterial and fungal communities to different fertilization regimes and seasons in *P. bournei* plantation forests. Firstly, organic fertilizer and compound microbial fertilizer changed, the composition of rhizosphere soil bacterial and fungal communities, respectively, and fungi were more sensitive to seasonal fluctuations than bacterial communities. Organic fertilizer management was more conducive to the turnover of both bacterial and fungal communities. Both bacterial and fungal community assembly processes were dominated by stochastic processes, with ecological drift controlling the assembly of fungal communities and dispersal limitation governing the assembly of bacterial communities. Our findings highlight the divergent responses of rhizosphere soil bacterial and fungal community assemblages and interactions with fertilizer regimes. These findings have significant implications for the sustainable management of *P. bournei* plantations. More broadly, research is also needed to determine the mechanism and response of rhizosphere microbiota under long-term fertilization application regimes.

## Supplementary information

ESM 1

## Data Availability

All data generated or analyzed during this study are included in this published article (and its supplementary information files).
