# Supplementary material for: Fertilization regime changes rhizosphere microbial community assembly and interaction in Phoebe bournei plantations
Source: Appl Microbiol Biotechnol. 2024 Jul 12;108:417. doi: 10.1007/s00253-024-13106-5 (PMC11245453; doi:10.1007/s00253-024-13106-5)
Supplement: Supplementary file 1 [file 253_2024_13106_MOESM1_ESM.pdf]

Supplemental Material

Title: Fertilization regime changes rhizosphere microbial community assembly and interaction in *Phoebe bournei* plantations

Haoyu Yan[1†], Yang Wu[1†], Gongxiu He[1], Shizhi Wen[1], Lili Yang[1*], Li Ji[1*]

[1]School of Forestry, Central South University of Forestry and Technology, 410004 Changsha, P.R. China

* Correspondence:

Lili Yang, znl_yll@163.com, School of Forestry, Central South University of Forestry and Technology, 410004 Changsha, P.R. China;

Li Ji, jl917@csuft.edu.cn, School of Forestry, Central South University of Forestry and Technology, 410004 Changsha, P.R. China; Tel: (+86) 130 8681 2452

†These authors contributed equally to this work.

Journal: Applied Microbiology and Biotechnology

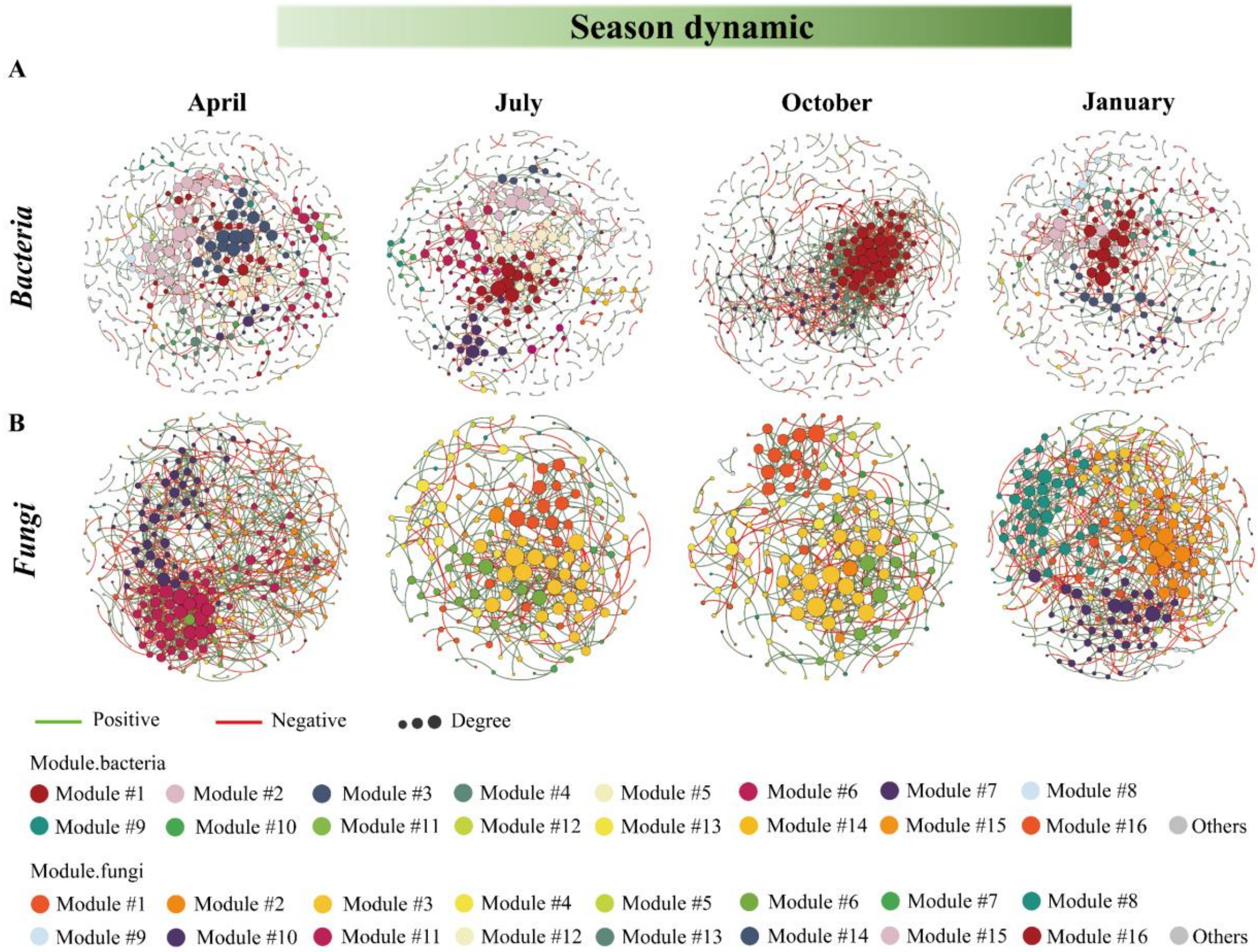

Fig. S1 Modular networks of bacteria (A) and fungi (B) across seasons. Node colors represent different modules. The connections denote strong (Spearman's $\rho > 0.6$) and significant ($P < 0.01$) correlations.

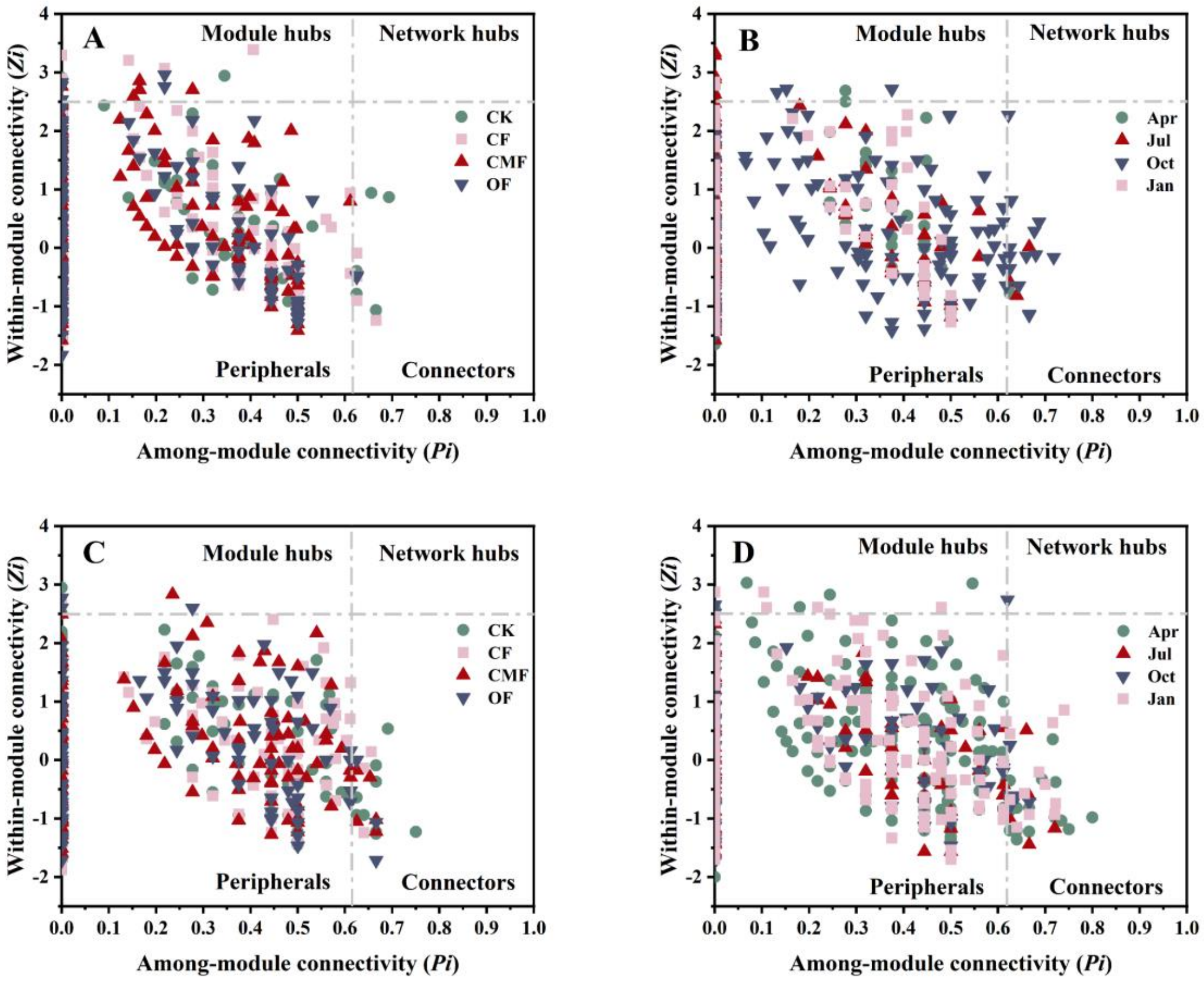

Fig. S2 Topological roles of OTUs in the soil bacterial (A, B) and fungal (C, D) co-occurrence networks under fertilizer regimes across seasons as indicated by the *Zi*-*Pi* plot.

Fertilizer regime

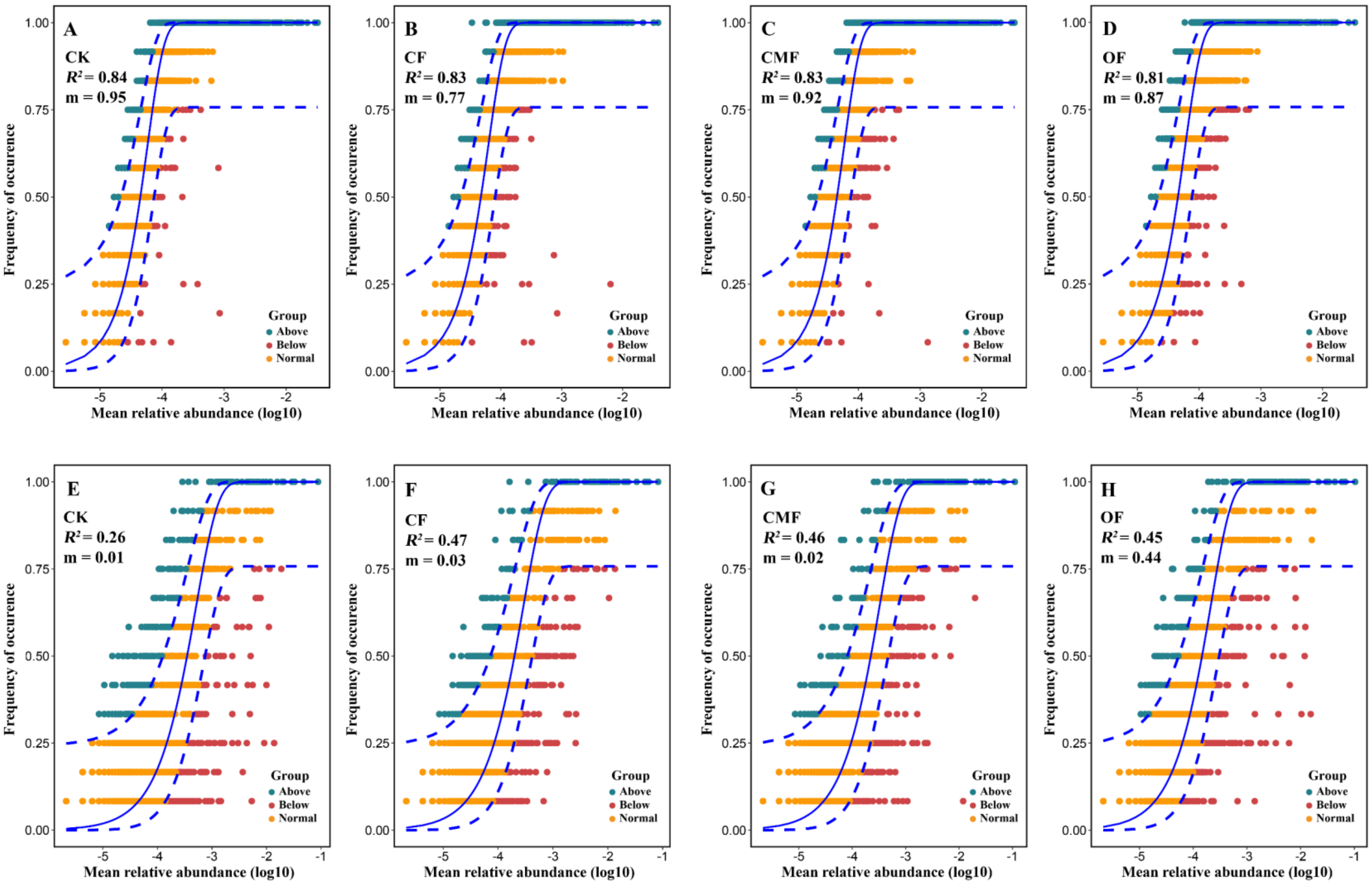

Season dynamic

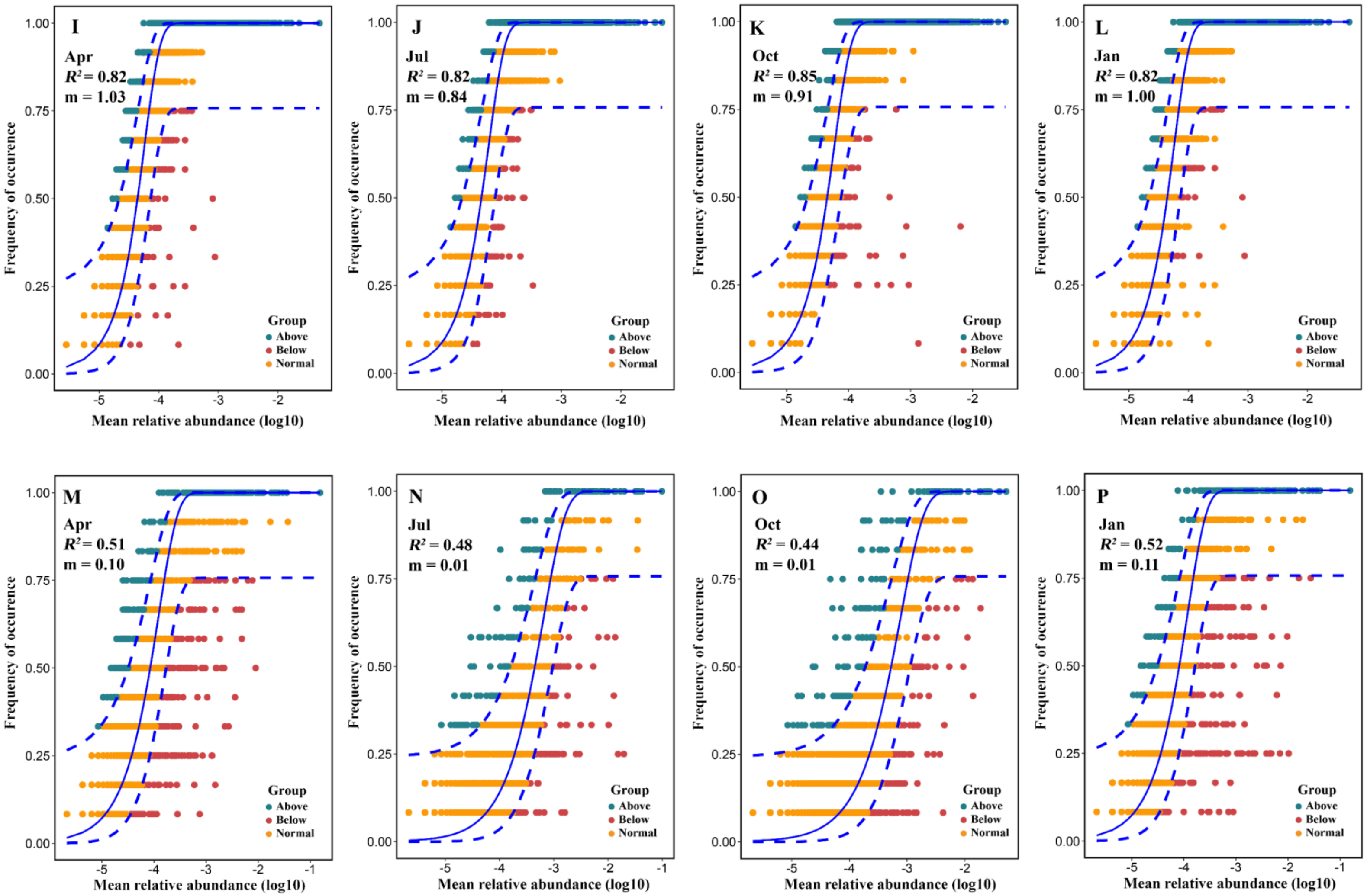

Fig. S3 Evaluation of the soil microbial community assembly process. The rhizosphere soil bacterial (A, B, C, D) and fungal (E, F, G, H) community assemblies under fertilizer regimes were analyzed using the neutral model. The rhizosphere soil bacterial (I, G, K, L) and fungal (M, N, O, P) community assemblies across seasons were analyzed using the neutral model. The solid blue line in the figure represents the most suitable neutral model, while the dotted line represents the 95% confidence interval around the best-fit neutral model. The OTUs within the confidence interval (represented by yellow points) follow a neutral process, the blue points indicate OTUs with a higher frequency than that predicted by the model, and the red points indicate OTUs with a lower frequency than that predicted by the model. R^2 represents the overall goodness of fit of the neutral community model. Higher R^2 indicates that the closer the model is to neutral, i.e., the community is more influenced by stochastic processes and less influenced by deterministic processes; m quantifies the metacommunity size times immigration, and the smaller the value of m indicates that the spread of species in the whole community is more restricted, and conversely, the higher the value of m indicates that the species are less restricted by the spread.

Table S1 Results of two-way ANOVA analysis considering the effects of fertilizers and seasons on bacterial communities.

Bacteria		Fertilizer	Season	Fertilizer × Season
Chemoheterotrophy	*F*	6.580	31.322	1.193
	P	0.001	<0.001	0.333
Aerobic_chemoheterotrophy	*F*	2.256	13.956	1.368
	P	0.101	<0.001	0.243
Cellulolysis	*F*	31.567	4.935	10.301
	P	<0.001	0.006	<0.001
Nitrogen_fixation	*F*	7.536	48.870	1.549
	P	0.001	<0.001	0.173
Animal_parasites_or_symbionts	*F*	3.574	9.522	0.721
	P	0.025	<0.001	0.686
Intracellular_parasites	*F*	12.192	5.781	1.867
	P	<0.001	0.003	0.094
Ureolysis	*F*	5.201	25.539	3.975
	P	0.005	<0.001	0.002
Invertebrate_parasites	*F*	2.040	16.623	1.272
	P	0.128	<0.001	0.289
Denitrification	*F*	3.423	6.787	1.754
	P	0.029	0.001	0.117
Sulfur_respiration	*F*	1.000	1.000	1.000
	P	0.405	0.405	0.460
Others	*F*	2.900	6.183	2.560
	P	0.050	0.002	0.024

Note: The *F*-value is used to evaluate the difference between groups. The *F*-value indicates the significance of the entire fitting equation. The larger the *F* is, the more significant the equation is and the better the fitting degree is. The *P*-value represents the level of significance in the hypothesis test. If $P < 0.01$, it means that the decision result is very significant and rejects the assumed parameter values. If $0.01 < P < 0.05$, it indicates that the decision result is more significant and rejects the assumed parameter value. If $P > 0.05$, it indicates that the results are more inclined to accept the assumed parameter values, and the difference level is not significant.

Table S2 Results of two-way ANOVA analysis considering the effects of fertilizers and seasons on fungal functional communities.

Fungi		Fertilizer	Season	Fertilizer × Season
Animal pathogen	*F*	2.389	2.586	3.044
	P	0.087	0.070	0.010
Plant pathogen	*F*	2.878	1.955	1.344
	P	0.051	0.141	0.254
Endophyte	*F*	0.660	47.857	1.241
	P	0.583	<0.001	0.306
Fungal parasite	*F*	6.189	111.081	4.500
	P	0.002	<0.001	0.001
Arbuscular mycorrhizal	*F*	10.699	51.945	8.096
	P	<0.001	<0.001	<0.001
Ectomycorrhizal	*F*	12.422	5.459	8.732
	P	<0.001	0.004	<0.001
Endomycorrhizal	*F*	12.422	5.459	8.732
	P	<0.001	0.004	<0.001
Epiphyte	*F*	0.965	13.546	0.836
	P	0.421	<0.001	0.589
Lichenized	*F*	6.220	4.182	3.306
	P	0.002	0.013	0.006
Dung saprotroph	*F*	1.217	4.220	1.087
	P	0.319	0.013	0.399
Litter saprotroph	*F*	0.971	5.300	2.735
	P	0.419	0.004	0.017
Soil saprotroph	*F*	0.497	42.153	5.348
	P	0.687	<0.001	<0.001
Undefined saprotroph	*F*	2.828	0.691	3.038
	P	0.054	0.564	0.01

Wood saprotroph	*F*	3.088	21.144	1.637
	P	0.041	<0.001	0.147

Note: The *F*-value is used to evaluate the difference between groups. The *F*-value indicates the significance of the entire fitting equation. The larger the *F* is, the more significant the equation is and the better the fitting degree is. The *P*-value represents the level of significance in the hypothesis test. If $P < 0.01$, it means that the decision result is very significant and rejects the assumed parameter values. If $0.01 < P < 0.05$, it indicates that the decision result is more significant and rejects the assumed parameter value. If $P > 0.05$, it indicates that the results are more inclined to accept the assumed parameter values, and the difference level is not significant.

Table S3 Keystone taxa in soil bacterial networks under fertilizer regimes across seasons.

	Group	OTU ID	Topological role	Phylum	Genus	Ecological function
**Fertilizer regime**	**CF**	OTU725	Connector	*Acidobacteriota*	*Candidatus_solibacter*	——
		OTU351	Connector	*Planctomycetota*	*Gemmataceae*	——
		OTU239	Connector	*Planctomycetota*	*Gemmataceae*	——
		OTU1343	Connector	*RCP2-54*	*RCP2-54*	——
		OTU4353	Module hub	*Proteobacteria*	*Xanthobacteraceae*	——
		OTU793	Module hub	*Verrucomicrobiota*	*Candidatus_udaeobacter*	——
		OTU1164	Module hub	*Proteobacteria*	*Elsterales*	——
		OTU210	Module hub	*Chloroflexi*	*Ktedonobacteraceae*	——
		OTU80	Module hub	*Chloroflexi*	*B12-WMSP1*	——
		OTU5163	Module hub	*WPS-2*	*WPS-2*	——
		OTU2733	Module hub	*Acidobacteriota*	*Subgroup_2*	——
	**CK**	OTU2528	Connector	*Proteobacteria*	*Elsterales*	——
		OTU649	Connector	*Actinobacteriota*	*Actinospica*	Aerobic_chemoheterotrophy; Chemoheterotrophy
		OTU3478	Connector	*Proteobacteria*	*Elsterales*	——
		OTU5002	Connector	*Actinobacteriota*	*Acidothermus*	Cellulolysis; Aerobic_chemoheterotrophy; Chemoheterotrophy
		OTU126	Connector	*Actinobacteriota*	*IMCC26256*	——
		OTU3637	Module hub	*Proteobacteria*	*Roseiarcus*	——
		OTU4528	Module hub	*Acidobacteriota*	*Acidobacteriales*	——
		OTU1244	Module hub	*Myxococcota*	*Myxococcota*	——
		OTU4411	Module hub	*Acidobacteriota*	*Subgroup_2*	——

		OTU2322	Module hub	*Actinobacteriota*	*IMCC26256*	——
	CMF	OTU2766	Module hub	*Acidobacteriota*	*Acidobacteriales*	——
		OTU4023	Module hub	*Acidobacteriota*	*Acidobacteriaceae_subgroup_1*	——
		OTU5163	Module hub	*WPS-2*	*WPS-2*	——
		OTU1317	Module hub	*Proteobacteria*	*Bradyrhizobium*	Aerobic_chemoheterotrophy; Chemoheterotrophy; Nitrogen_fixation
		OTU2322	Module hub	*Actinobacteriota*	*IMCC26256*	——
		OTU2594	Module hub	*Acidobacteriota*	*Acidobacteriales*	——
	OF	OTU3773	Module hub	*Myxococcota*	*Pajaroellobacter*	——
		OTU4986	Module hub	*Planctomycetota*	*Gemmataceae*	——
		OTU3617	Module hub	*Acidobacteriota*	*Acidobacteriales*	——
		OTU30	Module hub	*Chloroflexi*	*TK10*	——
		OTU22	Module hub	*Acidobacteriota*	*Subgroup_2*	——
		OTU4366	Module hub	*Acidobacteriota*	*Subgroup_2*	——
		OTU87	Connector	*Chloroflexi*	*Ktedonobacteraceae*	——
		OTU1543	Connector	*Proteobacteria*	*Xanthobacteraceae*	——
**Season**	**April**	OTU1657	Connector	*GAL15*	*GAL15*	——
		OTU558	Connector	*Proteobacteria*	*Caulobacteraceae*	——
		OTU1857	Module hub	*Chloroflexi*	*AD3*	——
		OTU273	Module hub	*Planctomycetota*	*Gemmataceae*	——
		OTU374	Module hub	*Chloroflexi*	*JG30a-KF-32*	——
		OTU2199	Module hub	*Acidobacteriota*	*Acidobacteriales*	——
		OTU418	Module hub	*Planctomycetota*	*Gemmataceae*	——
	**July**	OTU514	Module hub	*Actinobacteriota*	*Acidothermus*	Cellulolysis; Aerobic_chemoheterotrophy; Chemoheterotrophy
		OTU345	Module hub	*Verrucomicrobiota*	*Candidatus_udaeobacter*	——
		OTU1884	Module hub	*Acidobacteriota*	*Subgroup_2*	——

	OTU597	Module hub	*Actinobacteriota*	*Acidothermus*	Cellulolysis; Chemoheterotrophy; Aerobic_chemoheterotrophy
	OTU2049	Module hub	*Acidobacteriota*	*Subgroup_2*	——
	OTU2884	Module hub	*Acidobacteriota*	*Acidobacteriales*	——
**October**	OTU157	Connector	*Bacteroidota*	*Puia*	——
	OTU413	Connector	*Proteobacteria*	*Xanthobacteraceae*	——
	OTU2992	Connector	*Chloroflexi*	*JG30-KF-AS9*	——
	OTU1647	Connector	*Myxococcota*	*Polyangiaceae*	——
	OTU3400	Connector	*Proteobacteria*	*WD260*	——
	OTU4353	Connector	*Proteobacteria*	*Xanthobacteraceae*	——
	OTU1727	Connector	*Chloroflexi*	*G12-WMSP1*	——
	OTU3593	Connector	*Acidobacteriota*	*Acidipila*	——
	OTU2126	Connector	*Actinobacteriota*	*Gaiellales*	——
	OTU725	Connector	*Acidobacteriota*	*Candidatus_solibacter*	——
	OTU558	Connector	*Proteobacteria*	*Caulobacteraceae*	——
	OTU5707	Connector	*Actinobacteriota*	*Acidimicrobiia*	——
	OTU5772	Connector	*Actinobacteriota*	*Conexibacter*	——
	OTU4411	Module hub	*Acidobacteriota*	*Subgroup_2*	——
	OTU5586	Module hub	*Acidobacteriota*	*Subgroup_2*	——
	OTU3786	Module hub	*Acidobacteriota*	*Acidobacteriales*	——
**January**	OTU1748	Module hub	*WPS-2*	*WPS-2*	——
	OTU3150	Module hub	*Acidobacteriota*	*Occallatibacter*	——

Table S4 Keystone taxa in soil fungal networks under fertilizer regimes across seasons.

	Group	OTU ID	Topological role	Phylum	Genus	Guilds	Possibility
**Fertilizer regime**	**CK**	OTU767	Connector	*Ascomycota*	*Sordariomycetes*	-	-
		OTU289	Connector	*Ascomycota*	*Ascomycota*	-	-
		OTU311	Connector	*Basidiomycota*	*Solicoccozyma*	-	-
		OTU944	Connector	*Unclassified_fungi*	*Unclassified_fungi*	-	-
		OTU321	Connector	*Chytridiomycota*	*Chytridiomycota*	-	-
		OTU4095	Connector	*Unclassified_fungi*	*Unclassified_fungi*	-	-
		OTU1397	Connector	*Ascomycota*	*GS33*	-	-
		OTU2067	Connector	*Ascomycota*	*Ascomycota*	-	-
		OTU2439	Connector	*Ascomycota*	*Neopestalotiopsis*	-	-
		OTU812	Connector	*Ascomycota*	*Alternaria*	Animal pathogen<br>Endophyte<br>Plant pathogen<br>Wood saprotroph	Possible
		OTU1518	Module hub	*Glomeromycota*	*Glomeromycota*	Arbuscular mycorrhizal	Highly probable
		OTU2172	Module hub	*Glomeromycota*	*Glomerales*	Arbuscular mycorrhizal	Highly probable
	**CF**	OTU1297	Connector	*Unclassified_fungi*	*Unclassified_fungi*	-	-
		OTU3969	Connector	*Ascomycota*	*Ascomycota*	-	-
		OTU3175	Connector	*Ascomycota*	*Penicillifer*	Undefined saprotroph	Possible
		OTU65	Connector	*Glomeromycota*	*Glomeromycota*	Arbuscular mycorrhizal	Highly probable
		OTU3054	Module hub	*Unclassified_fungi*	*Unclassified_fungi*	-	-
		OTU1518	Module hub	*Glomeromycota*	*Glomeromycota*	Arbuscular mycorrhizal	Highly probable
	**CMF**	OTU1317	Connector	*Ascomycota*	*Scytalidium*	Wood saprotroph	Probable

		OTU3940	Connector	*Ascomycota*	*Chaetosphaeria*	Endophyte<br>Litter saprotroph<br>Wood saprotroph	Probable
		OTU1460	Connector	*Ascomycota*	*Ochroconis*	Undefined saprotroph	Probable
		OTU578	Connector	*Glomeromycota*	*Glomerales*	Arbuscular mycorrhizal	Highly probable
		OTU1020	Connector	*Glomeromycota*	*Glomeromycota*	Arbuscular mycorrhizal	Highly probable
		OTU280	Module hub	*Ascomycota*	*Aspergillaceae*	Undefined saprotroph	Possible
		OTU4363	Connector	*Mortierellomycota*	*Mortierella*	Endophyte<br>Litter saprotroph<br>Soil saprotroph<br>Undefined saprotroph	Possible
		OTU813	Connector	*Glomeromycota*	*Glomeraceae*	Arbuscular mycorrhizal	Highly probable
	**OF**	OTU1526	Connector	*Unclassified_fungi*	*Unclassified_fungi*	-	-
		OTU3858	Module hub	*Unclassified_fungi*	*Unclassified_fungi*	-	-
		OTU3940	Module hub	*Ascomycota*	*Chaetosphaeria*	Endophyte<br>Litter saprotroph<br>Wood saprotroph	Probable
		OTU3695	Module hub	*Unclassified_fungi*	*Unclassified_fungi*	-	-
**Season**	**April**	OTU2649	Connector	*Unclassified_fungi*	*Unclassified_fungi*	-	-
		OTU47	Connector	*Ascomycota*	*Humicolopsis_f__Pezizomycotina_fam_Incertae_sedis*	-	-
		OTU2747	Connector	*Unclassified_fungi*	*Unclassified_fungi*	-	-
		OTU3318	Connector	*Ascomycota*	*Fusarium*	Animal pathogen<br>Endophyte<br>Lichen parasite<br>Plant pathogen<br>Soil saprotroph<br>Wood saprotroph	Possible
		OTU647	Connector	*Ascomycota*	*Purpureocillium*	Fungal parasite	Probable

	OTU259	Connector	*Ascomycota*	*Fusidium*	Undefined saprotroph	Probable
	OTU1492	Connector	*Ascomycota*	*Capnodiales*	-	-
	OTU3530	Connector	*Ascomycota*	*Calyptrozyma*	Ectomycorrhizal<br>Fungal parasite<br>Plant pathogen<br>Wood saprotroph	Probable
	OTU630	Connector	*Ascomycota*	*Ascomycota*	-	-
	OTU1705	Connector	*Ascomycota*	*Gliocladiopsis*	Undefined saprotroph	Probable
	OTU453	Connector	*Ascomycota*	*Ascomycota*	-	-
	OTU1355	Connector	*Unclassified_fungi*	*Unclassified_fungi*	-	-
	OTU1212	Connector	*Ascomycota*	*Archaeorhizomycetes*	-	-
	OTU337	Connector	*Ascomycota*	*Sordariomycetes*	-	-
	OTU3531	Connector	*Unclassified_fungi*	*Unclassified_fungi*	-	-
	OTU1515	Module hub	*Unclassified_fungi*	*Unclassified_fungi*	Undefined saprotroph	Probable
	OTU70	Module hub	*Ascomycota*	*Penicillium*	Undefined saprotroph	Possible
	OTU2436	Module hub	*Ascomycota*	*Ascomycota*	-	-
	OTU1032	Module hub	*Mucoromycota*	*GS23*	-	-
	OTU1420	Module hub	*Ascomycota*	*Ascomycota*	-	-
**July**	OTU45	Connector	*Ascomycota*	*Aspergillaceae*	Undefined saprotroph	Possible
	OTU173	Connector	*Unclassified_fungi*	*Unclassified_fungi*	-	-
	OTU950	Connector	*Unclassified_fungi*	*Unclassified_fungi*	-	-
	OTU498	Connector	*Glomeromycota*	*Glomerales*	Arbuscular mycorrhizal	Highly probable
	OTU4045	Connector	*Ascomycota*	*Arnium*	Dung saprotroph<br>Undefined saprotroph	Highly probable
**October**	OTU4225	Connector	*Basidiomycota*	*Saitozyma*	Fungal parasite<br>Undefined saprotroph	Possible

	OTU624	Connector	*Mortierellomycota*	*Mortierella*	Endophyte<br>Litter saprotroph<br>Soil saprotroph<br>Undefined saprotroph	Possible
	OTU1113	Connector	*Ascomycota*	*Venturiales*	-	-
	OTU1411	Connector	*Ascomycota*	*Penicillium*	Undefined saprotroph	Possible
	OTU115	Connector	*Unclassified_fungi*	*Unclassified_fungi*	-	-
	OTU1794	Connector	*Ascomycota*	*GS34*	-	-
	OTU957	Network hub	*Unclassified_fungi*	*Unclassified_fungi*	-	-
	OTU258	Module hub	*Ascomycota*	*Ascomycota*	-	-
	OTU1421	Module hub	*Glomeromycota*	*Glomeromycota*	Arbuscular mycorrhizal	Highly probable
**January**	OTU311	Connector	*Basidiomycota*	*Solicoccozyma*	-	-
	OTU845	Connector	*Basidiomycota*	*Thanatephorus*	Plant pathogen	Probable
	OTU3858	Connector	*Unclassified_fungi*	*Unclassified_fungi*	-	-
	OTU132	Connector	*Basidiomycota*	*Ceratobasidiaceae*	Endomycorrhizal<br>Plant pathogen<br>Undefined saprotroph	Possible
	OTU2787	Connector	*Ascomycota*	*Penicillium*	Undefined saprotroph	Possible
	OTU1565	Connector	*Ascomycota*	*Scytalidium*	Wood saprotroph	Probable
	OTU1072	Connector	*Ascomycota*	*Sordariomycetes*	-	-
	OTU7	Connector	*Basidiomycota*	*Agaricales*	-	-
	OTU3596	Connector	*Ascomycota*	*Ascomycota*	-	-
	OTU1179	Connector	*Unclassified_fungi*	*Unclassified_fungi*	-	-
	OTU977	Connector	*Unclassified_fungi*	*Unclassified_fungi*	-	-
	OTU1609	Module hub	*Glomeromycota*	*Glomeromycota*	Arbuscular mycorrhizal	Highly probable
	OTU154	Module hub	*Basidiomycota*	*Hannaella*	Fungal parasite<br>Undefined saprotroph	Possible
	OTU33	Module hub	*Basidiomycota*	*Clavaria*	Undefined saprotroph	Probable
	OTU439	Module hub	*Ascomycota*	*Ascomycota*	-	-

OTU597	Module hub	*Ascomycota*	*Ascomycota*	-	-
